# Bispecific Antibodies in Hematological Malignancies: A Scoping Review

**DOI:** 10.3390/cancers15184550

**Published:** 2023-09-14

**Authors:** Mohamed H. Omer, Areez Shafqat, Omar Ahmad, Khaled Alkattan, Ahmed Yaqinuddin, Moussab Damlaj

**Affiliations:** 1 School of Medicine, Cardiff University, Cardiff CF14 4YS, UK; 2 College of Medicine, Alfaisal University, Riyadh 11533, Saudi Arabia; ashafqat@alfaisal.edu (A.S.); oahmad@alfaisal.edu (O.A.); kkattan@alfaisal.edu (K.A.); ayaqinuddin@alfaisal.edu (A.Y.); 3 Department of Hematology & Oncology, Sheikh Shakhbout Medical City, Abu Dhabi P.O. Box 11001, United Arab Emirates; mdamlaj@gmail.com; 4 College of Medicine, Khalifa University, Abu Dhabi P.O. Box 127788, United Arab Emirates

**Keywords:** bispecific antibody, antibodies, CAR-T, lymphoma, leukemia, multiple myeloma, hematological cancer

## Abstract

**Simple Summary:**

Bispecific T-cell engagers (BiTEs) and bispecific antibodies (BiAbs) have emerged as novel therapeutic modalities in the treatment of advanced hematological malignancies. BiTEs and BiAbs redirect T cells to attack tumors and facilitate T-cell-mediated cell death. Blinatumomab was the first BiTE to display proof-of-concept with its remarkable contribution towards the treatment of acute lymphoblastic leukemia. Nearly a decade later, several BiTEs/BiAbs targeting a range of tumor-associated antigens have transpired in the treatment of multiple myeloma, non-Hodgkin’s lymphoma, acute myelogenous leukemia, and acute lymphoblastic leukemia. This review summarizes the most recent evidence emerging from clinical trials regarding the use of BiAbs and BiTEs in hematological malignancies whilst highlighting the limitations of these therapeutic options and providing practical insights towards overcoming these limitations.

**Abstract:**

Bispecific T-cell engagers (BiTEs) and bispecific antibodies (BiAbs) have revolutionized the treatment landscape of hematological malignancies. By directing T cells towards specific tumor antigens, BiTEs and BiAbs facilitate the T-cell-mediated lysis of neoplastic cells. The success of blinatumomab, a CD19xCD3 BiTE, in acute lymphoblastic leukemia spearheaded the expansive development of BiTEs/BiAbs in the context of hematological neoplasms. Nearly a decade later, numerous BiTEs/BiAbs targeting a range of tumor-associated antigens have transpired in the treatment of multiple myeloma, non-Hodgkin’s lymphoma, acute myelogenous leukemia, and acute lymphoblastic leukemia. However, despite their generally favorable safety profiles, particular toxicities such as infections, cytokine release syndrome, myelosuppression, and neurotoxicity after BiAb/BiTE therapy raise valid concerns. Moreover, target antigen loss and the immunosuppressive microenvironment of hematological neoplasms facilitate resistance towards BiTEs/BiAbs. This review aims to highlight the most recent evidence from clinical trials evaluating the safety and efficacy of BiAbs/BiTEs. Additionally, the review will provide mechanistic insights into the limitations of BiAbs whilst outlining practical applications and strategies to overcome these limitations.

## 1. Introduction

T-cell-redirecting strategies have emerged as highly promising therapeutic modalities for the treatment of hematological malignancies. Notably, two approaches, namely chimeric antigen receptor T cells (CAR-T) and bispecific antibodies (BiAbs), have shown remarkable efficacy in the treatment of hematological malignancies. CAR-T cells have revolutionized the management of relapsed and refractory hematological malignancies like multiple myeloma (MM), non-Hodgkin’s lymphoma (NHL), and acute lymphoblastic leukemia (ALL) [1,2,3,4]. Despite CAR-T therapy’s remarkable success, its lengthy engineering process—spanning approximately 6–8 weeks—can render some patients with advanced disease ineligible for this therapy [5]. Furthermore, CAR-T therapy is often associated with multiple end-organ toxicities, including severe neurotoxicity and cytokine release syndrome (CRS), which may limit its utility, especially in patients with a lower performance status or other comorbidities [6].

On the other hand, BiAbs and bispecific T-cell engagers (BiTEs) offer the T-cell-redirecting capabilities of CAR-T as an off-shelf therapy whilst eliminating the logistical and time constraints associated with CAR-T delivery. Additionally, BiAbs and BiTEs appear to have a more favorable safety profile, with lower incidences of CRS and neurotoxicity than CAR-T therapy [7].

The pioneering success of blinatumomab as the first BiTE demonstrated proof-of-concept evidence with its remarkable contribution towards the treatment of ALL [8]. Nearly a decade later, several BiAbs and BiTEs have emerged in the therapeutic landscape of hematological neoplasms. This review summarizes the latest clinical trial evidence regarding the use of BiAbs and BiTEs in hematological malignancies. Furthermore, it aims to highlight the limitations associated with these therapeutic options and provide practical insights towards overcoming these limitations.

## 2. The Biology of Bispecific Antibodies

Given that our focus is summarizing clinical data on the use of BiAbs in hematological malignancies, a detailed discussion of the basic science of these drugs is beyond the scope of this review and we direct readers towards other reviews that have explored this area in greater detail [9,10].

### 2.1. Mechanism of Action

CAR-T therapy involves engineering a T cell to express a chimeric antigen receptor (CAR) specific to a tumor-associated antigen. Administering CAR-T cells would hence augment the anti-tumor immune response. BiAbs achieve a similar goal by containing two binding sites, enabling them to bind two epitopes on the same antigen or two different antigens [10,11]. One arm of the BiAb binds to the target tumor-associated antigen and the other simultaneously binds CD3 on the surfaces of CD4+ helper T cells and CD8+ cytotoxic T cells, resulting in the formation of an immunological synapse that activates T cells without the need for T cell recognition of the MHC/antigen complex on tumor cells [10]. Activated T cells release perforin and granzyme, resulting in the T-cell-dependent killing of tumor cells via apoptosis. While BiAbs are a broad category of antibodies that target two antigens or epitopes, the specific class of BiAbs that form immunological synapses between T cells and tumor cells are called bispecific T-cell engagers (BiTEs). The BiAbs discussed in this review are mostly BiTEs, and hence the two terms will be used interchangeably.

### 2.2. Resistance Mechanisms

Like other modalities of cancer treatment, tumors can become resistant to BiAbs/BiTEs and consequently impair therapeutic efficacy. The administration of BiAbs imposes significant selection pressures on tumor clones expressing the target tumor-associated antigen, but inadvertently confer a selective advantage to sub-clones lacking the target antigen, resulting in their expansion and resistance [12]. To counter this, combinatorial strategies of administering multiple BiAbs or trispecific antibodies that target an additional tumor antigen have been explored [12,13,14]. Additionally, specific genetic abnormalities in AML and ALL have been associated with an inferior response to BiAbs/BiTEs, but the underlying mechanism by which an adverse cytogenetic profile modulates the therapy response is unclear [15,16,17]. Specific tumor cells may also alter their intracellular signaling pathways in response to T-cell-redirecting therapy, as one study showed that disrupted interferon-gamma signaling in HER2-positive tumor cells conferred resistance to killing by BiTE/CAR-redirected T cells [18]. Alternatively, features extrinsic to the tumor cell, such as the strong presence of regulatory T cells in the tumor microenvironment (TME), have been shown to modulate the therapeutic response to BiAbs/BiTEs in multiple myeloma and B-cell ALL [19,20,21,22]. Lastly, resistance may develop as a consequence of prior lines of cancer treatment itself, which can lead to a loss of T cell fitness and anti-tumor function [23]. Long-term administration of BiTEs can continually stimulate T cells, promoting their exhaustion and thereby promoting tumor survival [24,25]. The specific mechanisms at play in different types of hematological malignancies are discussed in their respective sections below.

## 3. Bispecific T-Cell Engagers and Antibodies in the Treatment of Multiple Myeloma

The introduction of protease inhibitors, immunomodulatory drugs (IMIDs), and anti-CD38 monoclonal antibodies has significantly improved multiple myeloma (MM) patient outcomes [26], but those with high-risk disease and adverse cytogenetic profiles often do not respond to these treatments [27]. Such patients, often termed ‘triple refractory’, exhibit poor survival outcomes [28]. In this regard, BiAbs and BiTEs have emerged as promising additions to the MM treatment landscape, particularly for triple-refractory MM.

A prime target for BiTE therapy in MM is the B-cell maturation antigen (BCMA). BCMA is selectively expressed on the surfaces of plasma cells and is associated with disease severity and unfavorable prognostic outcomes [29,30]. Teclistamab, a humanized IgG BCMA-targeting BiAb, was recently approved by the European Medicines Agency (EMA) and the Food and Drug Administration (FDA) for relapsed-refractory multiple myeloma (RRMM) [31]. The MajesTEC-1 clinical trial demonstrated that teclistamab had an overall response rate (ORR) of 63.0% and a complete response in 39.4% of 165 patients during an average follow-up of 14.1 months [32]. The median duration of response and progression-free survival was 18.4 months and 11.3 months, respectively. Adverse events included grade 1–2 CRS, cytopenias, and infections [32]. Elranatamab, another BCMA targeting IgG2A BiAb, has also shown promise in RRMM patients. It has obtained orphan drug designation by the EMA and FDA. The MagnetisMM-3 trial demonstrated an ORR of 61.0%, with a complete response achieved in 27.6% of 123 enrolled patients with triple-refractory MM during a median follow-up duration of 6.8 months [33]. CRS was the most common side effect, with an otherwise manageable safety profile [33]. The recent phase 2 LINKER-MM1 clinical trial explored the use of the anti-BCMA BiTE linvoseltamab in triple-refractory MM, reporting an ORR of 64% in patients receiving a higher dose (200 mg) compared to 50% for those on the lower dose (50 mg). Linvoseltamab demonstrated a tolerable safety profile, with CRS and infections as the most common adverse events [34]. ABBV-383, an anti-BCMA BiAb/BiTE, has the advantage of not requiring step-up dosing, making it easier to administer and monitor. In a phase 1 trial, ABBV-383 achieved an ORR of 57% and a complete response in 29% of 124 RRMM patients [35].

The use of BCMA-targeting therapeutic modalities in MM may result in either the decreased or complete loss of BCMA expression on MM cells and consequent antigen escape [36]. To address this issue, researchers have explored the targeting of additional antigens with BiTEs. G-protein-coupled receptor family C group 5 member D (GPRC5D), expressed on neoplastic MM cells, is one such target [37]. The MonumenTAL-1 phase 1 clinical trial reported that talquetamab, an IgG4 Fc BiAb directed against GPRC5D, demonstrated an ORR of 64–70% in 232 heavily pretreated RRMM patients [38]. The main adverse events associated with talquetamab were CRS in 77–80% of patients (primarily grades 1–2) and hematologic toxicity [38]. Talquetamab was additionally associated with unique adverse effects of skin and nail disorders, likely explained by the expression of GPRC5D in keratinized tissues and hair follicles [38]. However, the majority of these particular adverse events were well tolerated in the MonumenTAL-1 trial [37]. Another GPRC5D targeting BiTE, RG6234, demonstrated an ORR of 71.4% in RRMM patients during a phase 1 clinical trial [39]. This trial also evaluated the response in patients who received prior BCMA-targeting BiAb therapy, demonstrating an ORR of 55.6% in these patients [39].

The Fc Receptor Homolog 5 (FcRH5) and CD38 have been considered potential targets for BiAbs in MM [40]. Cevostamab, a humanized anti-FcRH5 IgG1 BiAb, elicited treatment responses in patients previously exposed to CAR-T (44.4%), BiAbs (33.3%), antibody–drug conjugates (50.0%), and BCMA-targeted therapy (36.4%) in an ongoing phase 1 study enrolling 160 RRMM patients with a manageable safety profile, with grade 1–2 CRS being the most common side effect, indicating its potential as a salvage therapy [41]. CD38, a transmembrane glycoprotein, is expressed on neoplastic plasma cells in MM and is a recognized component of the immunosuppressive TME [42]. ISB 1342, a CD3xCD38 BiAb/BiTE, demonstrated a manageable safety profile in 24 patients with RRMM during a phase 1 dose-escalation study [43]. The development of novel targets for BiAbs in multiple myeloma is still ongoing. Of particular interest is CD138, a transmembrane proteoglycan that is highly expressed on the surface of neoplastic plasma cells [44].

These findings indicate that monotherapy with BiAbs holds tremendous therapeutic potential in MM patients (Table 1). However, the ever-present risk of antigen escape may hinder the efficacy of BiAbs. Combinatorial approaches targeting multiple antigens simultaneously have been proposed to mitigate antigen escape. The RedirecTT-1 trial enrolled RRMM patients to receive teclistamab and talquetamab, simultaneously targeting BCMA and GPRC5D, respectively [45]. A total of 63 patients received this combination therapy, achieving an ORR of 84% across all dosages [45]. Moreover, the ORR at the recommended phase 2 regimen dose was 92%. CRS and cytopenias were the most common adverse events [45]. Another potential mechanism to improve responses to BiAbs is to upregulate target antigens to MM cells. For instance, inhibiting gamma-secretase, which cleaves BCMA and releases it into the circulation, by nirogacestat has been shown to increase the expression of BCMA on multiple myeloma cells [46]. Two ongoing phase 1 studies are investigating the safety of combining nirogacestat and anti-BCMA BiAbs (NCT04722146 and NCT05090566).

Besides antigen escape, the immunosuppressive TME in MM poses challenges to the efficacy of BiAb therapy. The immune microenvironment in MM is characterized by the infiltration of T-regs and the upregulation of programmed death ligand 1 (PD-L1) on MM cells [47,48,49]. The MajesTEC-1 trial demonstrated that exhausted CD8+ T cells coupled with greater levels of T-regs resulted in lower response rates and inferior outcomes to teclistamab [22]. Moreover, the immunosuppressive TME in MM progresses in correlation with the length of disease and exposure to multiple lines of therapy [50]. Mechanistically, patients at earlier stages of their disease have more functional CD8+ cytotoxic T cells along with reduced levels of immunosuppressive T-regs [50]. Hence, the earlier utilization of BiAbs/BiTEs may improve the tumor therapy response. Several clinical trials are currently underway to explore the role of BiAbs in earlier disease stages, particularly as an adjunct to control disease activity post-autologous stem cell transplantation (NCT05623020, NCT05552222, NCT05243797, NCT05317416).

**Figure 1:**
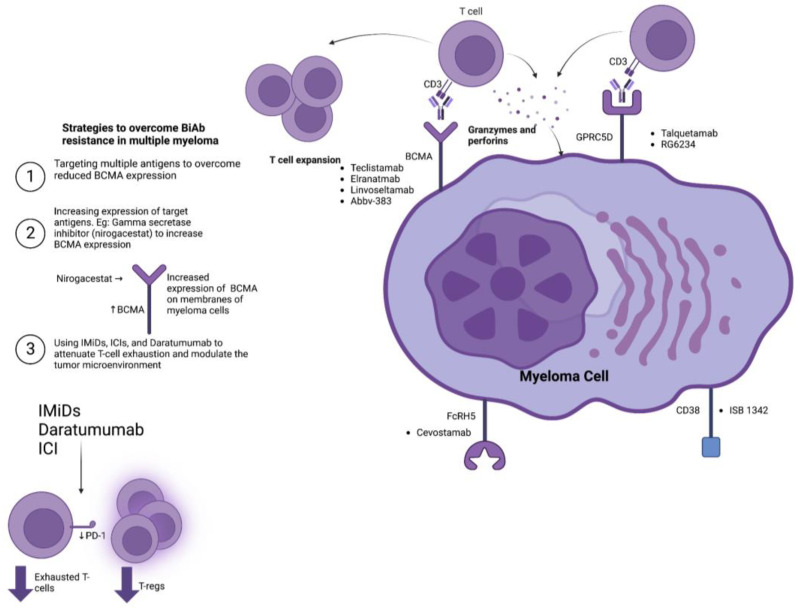
This figure depicts the main bispecific antibodies in ongoing clinical trials for the treatment of multiple myeloma. The bispecific antibodies are outlined according to their target myeloma-associated antigen, including BCMA, GPRC5D, FcRH5, and CD38. The figure also outlines the key strategies to overcome bispecific antibody resistance. One of the key strategies includes the synergistic combination of bispecific antibodies with other bispecific antibodies, monoclonal antibodies, or antibody–drug conjugates in order to target multiple antigens simultaneously. The second strategy to overcome resistance is relevant to the main class of bispecific antibodies in multiple myeloma, i.e., BCMA-targeting bispecific antibodies. This strategy involves using gamma-secretase inhibitors such as nirogacestat to prevent the cleavage of membrane-bound BCMA into soluble BCMA, thereby increasing the expression of BCMA on the surfaces of myeloma cells. Finally, the third strategy involves the use of immunomodulatory agents, immune checkpoint inhibitors, and daratumumab to modulate the tumor microenvironment and shift the repertoire of T cells by reducing the number of immunosuppressive regulatory T cells and exhausted T cells.

Reprogramming the TME to augment anti-tumor T-cell immunity may also improve MM responses to BiAb. In this context, IMiDs, such as lenalidomide and thalidomide, have also demonstrated the ability to enhance T-cell-directed responses against MM cells in vitro and in vivo [51]. Daratumumab, an anti-CD38 monoclonal antibody, can induce T cell expansion whilst skewing the repertoire of the TME T cells towards effector cytotoxic CD8+ T cells [52]. Clinical studies combining IMiDs and daratumumab with BiAbs are currently in their infancy, but preliminary results have shown promising outcomes. Combining teclistamab with daratumumab and lenalidomide achieved an ORR of 90% with tolerable safety profiles in a phase 1 trial [53]. The phase 1b TRIMM-2 trial combined teclistamab and daratumumab in RRMM patients and reported an ORR of 78% with manageable safety profiles [54]. Lastly, the use of immune checkpoint inhibitors, particularly agents targeting PD-L1/PD-1, can improve cytotoxic CD8+ T cell function; hence, combining ICIs with BiAbs may constitute another approach to improve responsiveness by modulating the TME of MM [55]. Figure 1 provides an overview of the BiAbs in MM and highlights the mechanisms of resistance towards them.

In summary, BiAbs represent an effective therapeutic approach for RRMM in terms of response rates and safety profiles. Overcoming antigen escape, either by combinatorial approaches employing mechanistically diverse BiAbs or pharmacologically upregulating the expression of target antigens, is an emerging area of investigation. Overcoming the immunosuppressive TME, either by earlier intervention or reprogramming it through IMiDs or ICIs, holds promise in terms of improving outcomes in RRMM patients.

## 4. Bispecific T-Cell Engagers and Antibodies in the Treatment of Acute Lymphoblastic Leukemia

Precursor B-cell acute lymphoblastic leukemia (B-ALL) is characterized by the malignant proliferation of B-lineage precursor cells in the bone marrow and peripheral blood [56]. While survival rates for adult and pediatric B-ALL patients have improved with the development of effective chemotherapeutic protocols and salvage therapies, approximately 10% of patients develop refractory disease, and there is a significant risk of relapse even after achieving initial remission—hence the need for novel therapeutic options that improve survival outcomes and facilitate minimal residual disease (MRD) clearance [57,58]. BiTEs constitute a promising strategy by reprogramming the immune system and directing T cells toward neoplastic progenitor B cells.

CD19, a critical mediator of B-cell signaling, is expressed on most cells of the B-lymphocyte lineage and is maintained during the neoplastic transition of precursor B cells in B-ALL [59]. This makes CD19 an attractive target antigen for bispecific antibody therapy. Blinatumomab is a BiTE that targets CD19 on neoplastic precursor B cells and CD3 expressed on T cells and pioneered the early development of BiAbs. The efficacy of blinatumomab was established through the phase 3 clinical trial TOWER, which compared blinatumomab to standard-of-care chemotherapy in the treatment of relapsed/refractory B-ALL [60]. The trial enrolled 405 patients, of which 271 patients received blinatumomab and 134 patients were given standard-of-care chemotherapy [60]. Blinatumomab demonstrated a significant improvement in overall survival (7.7 months vs. 4 months) along with an increase in the rates of complete remission (34% vs. 16%) [60]. However, despite achieving complete hematological remission in approximately 90% of patients, around 50% of adult patients with B-ALL still showed evidence of minimal residual disease positivity [61,62,63,64], which was strongly associated with a higher risk of relapse [65,66]. Consequently, blinatumomab was evaluated in a phase 2 clinical trial for the treatment of MRD-positive B-ALL in patients with complete hematological remission, achieving a complete MRD response in 78% of patients, leading to improved overall survival and relapse-free survival [67]. In pediatric B-ALL, phase 3 clinical trials in the pediatric population with relapsed/refractory B-ALL have demonstrated that blinatumomab is associated with improved MRD clearance and an improved likelihood of transition towards allogeneic stem cell transplantation [68,69]. The safety profile of blinatumomab is generally favorable, with primary toxicities including infection, hematologic toxicity, and neurotoxicity, and a lower incidence of CRS than other BiABs/BiTEs [60,70]. Moreover, CRS frequency can be reduced further with blinatumomab following premedication with dexamethasone and the implementation of step-up dosing. Although neurotoxicity with blinatumomab is more frequent when compared to other BiAbs/BiTEs, the clinical manifestations are transient in the majority of cases, and an improvement is noted swiftly following appropriate treatment and the interruption of blinatumomab treatment [71]. Based on these results, blinatumomab has received approval from the FDA/EMA for the treatment of relapsed/refractory B-ALL and B-ALL with MRD positivity despite complete hematological remission [72].

The success of blinatumomab has inspired investigations into its potential utility across different clinical presentations in B-ALL. Blinatumomab has been studied as an adjunct to consolidation chemotherapy in patients with MRD-negative B-ALL, demonstrating significant improvements in overall survival compared to standard consolidation alone [73]. It has also been recently evaluated in a phase 2 clinical trial in combination with induction chemotherapy in adults with Philadelphia chromosome-negative ALL, achieving MRD negativity in 92% of patients [74]. These findings suggest that blinatumomab may reduce the need for allogeneic stem cell transplantation in certain patient populations; however, more data are required from clinical trials along with greater follow-up times. Blinatumomab is currently being evaluated as a potential maintenance therapy post-allogeneic stem cell transplantation in B-ALL patients [74]. In Philadelphia chromosome t(9:22)-positive ALL, which has a poor prognosis with inferior treatment responsiveness to conventional chemotherapy, chemotherapy-free induction and consolidation regimens with blinatumomab and tyrosine kinase inhibitors (TKIs) have shown promising outcomes in two phase 2 clinical trials, indicating that this combination may potentially be superior to intensive chemotherapy, particularly in unfit patients [15,75,76,77].

**Figure 2:**
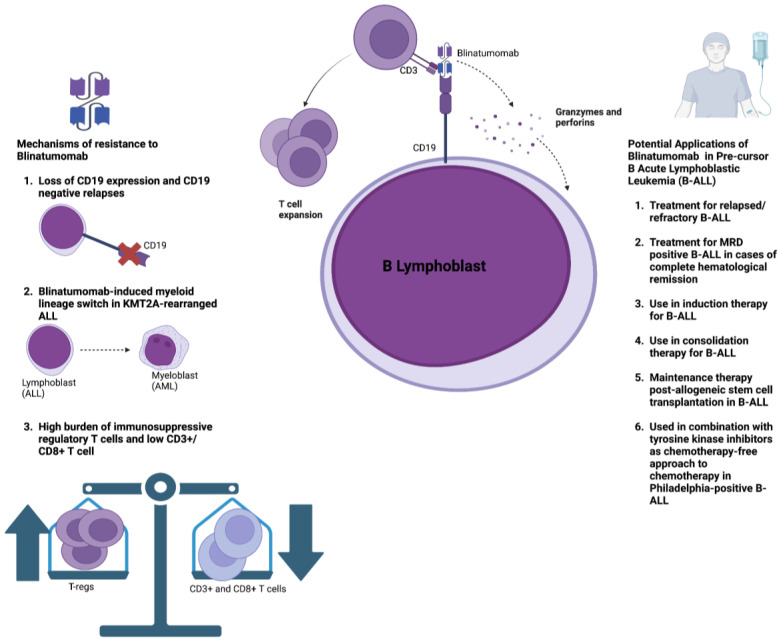
This figure outlines the mechanism of action of blinatumomab, the main bispecific T-cell engager utilized in the treatment of acute lymphoblastic leukemia. Blinatumomab binds to the CD19 antigen expressed on neoplastic B lymphoblasts along with the CD3 receptor expression on T cells, resulting in subsequent T-cell-mediated lysis of the leukemic cell. The figure also outlines some of the potential applications of blinatumomab therapy in acute lymphoblastic leukemia. This includes its use for relapsed/refractory disease, in addition to its incorporation in induction and consolidation regimens for different subtypes of acute lymphoblastic leukemia. The figure also illustrates the key mechanisms of resistance associated with blinatumomab therapy, including (1) antigen escape and loss of CD19 expression; (2) myeloid lineage switch after blinatumomab therapy, which has been reported in cases of KMT2A(MLL)-rearranged acute lymphoblastic leukemia, resulting in the development of acute myelogenous leukemia; (3) the immunosuppressive microenvironment in acute lymphoblastic leukemia is associated with an increased percentage of regulatory T cells along with a lower frequency of CD8+/CD3+ T cells, thereby facilitating resistance to blinatumomab.

In summary, revolutionary advances in B-ALL treatment have been made, spearheaded by the development of blinatumomab. Table 2 provides an overview of the main studies that outline the utility of blinatumomab in different patient populations. Nonetheless, resistance to blinatumomab poses a significant challenge to its efficacy (Figure 2). Loss of CD19 surface expression and subsequent antigen escape occurs in approximately 10–15% of patients who have relapsed following blinatumomab therapy [78,79,80]. Possible mechanisms underpinning the loss of CD19 expression include its alternative mRNA splicing, disrupted CD19 membrane trafficking, and the clonal expansion of leukemic cells that contain CD19 deletions [81,82]. Overcoming CD19 antigen loss mainly relies on identifying novel target antigens. CD22 is a novel target antigen that is expressed in the majority of leukemic blasts in B-ALL [83]. Combination therapies involving CD19 and CD22 CAR-T therapy have demonstrated promising preclinical potential and are currently being investigated in clinical trials [84,85,86]. The anti-CD22 antibody–drug conjugate inotuzumab ozogamicin (INO) has shown promising outcomes in the treatment of B-ALL, and combining INO with blinatumomab may represent a novel approach to combat antigen escape [87]. A recent phase 2 trial assessed the effect of INO with or without blinatumomab in combination with low-intensity chemotherapy amongst older adults with relapsed/refractory B-ALL, demonstrating promising outcomes regarding survival and disease clearance [88]. Another potential mechanism of resistance is the possibility of a myeloid lineage switch following blinatumomab therapy, particularly in KMT2A(MLL)-rearranged ALL, which may lead to the development of AML [89,90,91,92]. A recent preclinical study evaluated the possibility of combining anti-CD19 and anti-CD33 BiAbs to target tumor heterogeneity and prevent clonal escape [93].

Lastly, the immunosuppressive TME in ALL may promote resistance to blinatumomab [94]. A higher burden of T-regs has been associated with resistance to blinatumomab, whereas a greater presence of CD8+ effector and memory T cells and CD3+ T cells is associated with a better response to treatment [21,95]. B-ALL patients who do not respond to blinatumomab exhibit T-cell deficiency in the TME and higher levels of immune checkpoint molecules such as PD-1, TIM-3, and TIGIT compared to responders [95,96]. In agreement with these findings, a recent phase 2 clinical trial on patients with chronic lymphocytic leukemia (CLL) and Richter’s transformation to diffuse large B-cell lymphoma (DLBCL) showed that complete responders to blinatumomab expressed the lowest levels of PD-1, TIM-3, and TIGIT [97]. T-cell exhaustion may be related to exposure to multiple lines of cancer therapy before blinatumomab, as these agents are not typically used as first-line treatments, or from continuous exposure to blinatumomab, with the persistent T-cell stimulation causing subsequent exhaustion [24,25]. Accordingly, strategies to reprogram the immunosuppressive TME include treatment-free intervals, which can reduce T-cell exhaustion, and the use of ICIs such as nivolumab and pembrolizumab [24]. Results from early-stage clinical trials demonstrate that combining ICIs with blinatumomab is safe; however, efficacy results are still awaited [98,99].

## 5. Bispecific T-Cell Engagers and Antibodies in the Treatment of Non-Hodgkin’s Lymphoma

Non-Hodgkin’s lymphoma (NHL) encompasses a diverse group of lymphoproliferative neoplasms with varying grades of progression and severity [100]. Among the numerous NHL subtypes, indolent follicular lymphoma (FL) and diffuse large B-cell lymphoma (DLBCL) are the most common [100]. The introduction of the anti-CD20 monoclonal antibody rituximab has significantly improved the prognosis of B-cell NHL. However, a significant number of patients develop relapsed and/or refractory disease that does not respond to conventional chemotherapy [101]; hence, the need arises for novel treatment strategies, such as those that harness T-cell-mediated anti-neoplastic activity. CAR-T therapy has demonstrated remarkable efficacy in the treatment of relapsed/refractory NHL, but urgent intervention is required for patients with rapidly progressive disease [102].

BiAbs targeting multiple effector cell surface markers (CD3, CD16a, 4-1BBL, CD28, CD47) and B-cell antigens (CD19, CD20, CD22, CD37, CD79b) have been developed for NHL treatment [103]. CD20 is a critical B-cell surface antigen that is expressed on approximately 90% of malignant B cells but not on hematopoietic stem cells, minimizing the risk of myelosuppression [104,105]. These characteristics render CD20 an attractive target antigen for BiAbs in NHL [106]. Currently, several CD3xCD20 BiAbs, including glofitamab, mosunetuzumab, epcoritamab, odronextamab, and Igm-2323, have shown significant activity in the treatment of both indolent and aggressive NHL subtypes, including FL, DLBCL, transformed follicular lymphoma (tfFL), primary mediastinal large B-cell lymphoma (PMBCL), mantle cell lymphoma (MCL), and Richter’s transformation, in phase 1 and 2 clinical trials (Table 3). In patients with relapsed/refractory FL (RRFL), mosunetuzumab and odronextamab have demonstrated compelling efficacy, achieving complete response rates of 60% and 75%, respectively [107,108]. In DLBCL patients, glofitamab, odronextamab, and epcoritamab displayed similar complete response rates of 37–39% [109,110,111,112]. However, when considering the durability of responses, glofitamab showed better results, with 70% of patients still in complete remission after 18 months, compared to 48% with odronextamab [110,112].

Regarding adverse events, low-grade CRS is the most common side effect associated with CD20xCD3 antibodies, with grade ≥3 occurring rarely (Table 3). CRS events usually occur during the first cycle of treatment, and their severity can be attenuated by step-up dosing, premedication with steroids, and the administration of a B-cell-depleting agent [107,108,110,111,113,114]. Other adverse events, such as pyrexia, neutropenia, anemia, and electrolyte changes, are transient and clinically insignificant. Importantly, CD20xCD3 BiAbs are associated with a lower incidence and severity of immune effector cell-associated neurotoxicity syndrome (ICANS) than CAR-T therapy [110,115]. Furthermore, infections were common but varied between BiAbs, which could be attributed to different rates of neutropenia among BiAbs (38% with glofitamab vs. 21.7% with epcoritamab) [110,111]. Similarly, hypogammaglobulinemia associated with CD20xCD3 BiAbs may predispose patients to infection.

**Table 3:** Phase 1 and 2 studies evaluating the safety and efficacy of CD20xCD3 bispecific antibodies in different subtypes of non-Hodgkin’s lymphoma.

BiAB, Trial	BiAB Structure	N	Design	ORR, CR (%)	CRS (All Grade, ≥Grade 3) %	ICANS %	Infections %
Mosunetuzumab (Ph2, NCT02500407) [107]	IgG1, humanized	90	IV, 21-day cycles, step-up dosing (1/2/60/60 mg) then 30 mg onwards. Pts achieving a CR by cycle 8 completed treatment; those with a partial response or stable disease received 17 cycles total	RRFL 77.8, 60.0	44.0, 2.0	NR	NR
Odronextamab (Ph2, NCT03888105) [108]	Fully human IgG4-based	96	IV, 21-day cycles, step-up dosing in two regimens (1/20 mg or 0.7/4/20 mg) then 80 mg till cycle 4. Followed by 160 mg maintenance every 2 weeks till disease progression or unacceptable toxicity	RRFL 81.0, 75.0	51.0, 0.0	0.0 in 0.7/4/20 regimen 3.0 in 1/20	NR
Odronextamab (Ph2, NCT03888105) [112]	Fully human IgG4-based	121	IV, 21-day cycles, step-up dosing in two regimens (1/20 mg or 0.7/4/20 mg) then 160 mg till cycle 4. Followed by 320 mg maintenance every 2 weeks till disease progression or unacceptable toxicity	RR DLBCL 53.0, 37.0	53.0, 0.0	4.0 in 0.7/4/20 regimen 6.0 in 1/20	NR
Epcoritamab (Ph2, NCT03625037) [111]	IgG1, humanized	157	SQ, 28-day cycles, once weekly step-up doses in weeks 1–3 of cycle 1, then full doses once weekly through cycle 3, once every 2 weeks in cycles 4–9, and once every 4 weeks in cycle 10 and thereafter, until disease progression or unacceptable toxicity	RR DLBCL 63.0, 39.0	49.7, 2.5	6.4 (one death)	45.2
Glofitamab (Ph2, NCT03075696) [109]	2:1 configuration with bivalency to CD20	154	Pre-treatment with 1000 mg obinutuzumab, followed by IV glofitamab 7 days later, 21-day cycles, two step-up doses (2.5/10 mg) then 30 mg for 12 cycles.	RR DLBCL 58.0, 38.0	64.0, 4.0	8.0	59.0
Mosunetuzumab (Ph1/2, NCT02500407) [116]	IgG1, humanized	89	SQ, 21-day cycles, step-up dosing, 3 groups (5/15/45 mg, 5/45/45 mg, 5/45/90/90/45 mg) then 45 mg onwards. Pts achieving a CR by cycle 8 completed treatment; those with a partial response or stable disease received 17 cycles total	iNHL 82.0, 64.0 aNHL 36.0, 20.0	27.0, 0.0	3.0	14.0 grade 3/4
Igm-2323 (Ph1, NCT04082936) [117]	Ten binding domains for CD20; one binding domain for CD3	29	IV on days 1, 8, and 15 of 21-day cycles until disease progression	(FL *n* = 11) (DLBCL *n* = 13) (MCL *n* = 3) (MZL *n* = 2) 34.8, 21.7	20.7, NR	0.0	NR

BiAb = bispecific antibody. CRS = cytokine release syndrome. ICANS = immune effector cell-associated neurotoxicity syndrome. ORR = overall response rate. CR = complete response. NR = not reported. DLBCL = diffuse large B-cell lymphoma. iNHL = indolent NHL. aNHL = aggressive NHL. MCL = mantle cell lymphoma. MZL = marginal zone lymphoma. SQ = subcutaneous.

CD19 is another potential target antigen in NHL due to its ubiquitous expression on B cells, including neoplastic B cells [118]. CD19-targeting BiAbs, such as blinatumomab, AFM11, duvortuxizumab, and Tnb-486, have been evaluated in NHL treatment [119,120,121,122]. Blinatumomab’s success in the treatment of ALL paved the way for efforts to explore its efficacy in NHL. Blinatumomab has shown substantial efficacy for the treatment of NHL in phase I and II clinical trials but is associated with a high rate of potentially severe neurological events [119,123]. The high frequency of neurological events, coupled with blinatumomab’s narrow half-life, necessitating continuous infusions, has halted any further development in its use for NHL [119,123]. Similarly, phase I studies assessing AFM11 and duvortuxizumab were discontinued due to neurotoxicity concerns [120,122]. Tnb-486, a novel CD3xCD19 BiAb, demonstrated a complete response in 91% of RRFL patients in a phase 1 trial and had a lower incidence of ICANS and CRS compared to blinatumomab [119,121,123]. Mechanistically, the lower incidence of ICANS and CRS associated with Tnb-486 is likely due to its unique anti-CD3 moiety, designed to bind CD3 on T cells with low affinity, thereby attenuating the release of pro-inflammatory cytokines [124]. The higher incidence of ICANS observed with CD19xCD3 BiAbs when compared to CD3xCD20 BiAbs may be due to potential on-target off-tumor toxicity associated with targeting CD19, which is expressed on the pericytes and vascular smooth muscles that line the blood–brain barrier (BBB); therefore, the use of anti-CD19 BiAbs such as blinatumomab may impair the integrity of the BBB [125].

The CD20xCD3 BiAbs mosunetuzumab, glofitamab, and epcoritamab have received accelerated FDA approval for specific NHL subtypes due to their substantial efficacy. However, factors impairing the efficacy of CD20xCD3 BiAbs require further exploration. For example, antigen escape resulting from reduced CD20 expression has been observed in a significant number of NHL patients treated with rituximab and was associated with an inferior prognosis [126]. Loss of CD20 expression has also been associated, deemed to be a potential contributor towards resistance to the CD20xCD3 BiAb mosunetuzumab [121]. Upregulating CD20 expression on NHL cells may, therefore, constitute a potential mechanism to enhance the efficacy of CD20xCD3 BiAbs. In this regard, gemcitabine can upregulate CD20 on DLBCL cells in vitro, which enhances the antitumor activity of rituximab [127]. A recent phase Ib/II trial demonstrated that epcoritamab + GemOx (gemcitabine, oxaliplatin) in RR DLBCL displayed a higher ORR than epcoritamab monotherapy (92% vs. 63%) [111,124]. Moreover, ameliorating antigen escape through targeting multiple antigens simultaneously in NHL may provide a novel approach to enhance their efficacy [113,128]. The antibody–drug conjugate polatuzumab vedotin (PV) targets CD79b, an antigen that is expressed on the majority of malignant B cells in NHL [129]. Two phase Ib/II clinical trials assessing the efficacy and safety of glofitamab and mosunetuzumab in combination with PV have demonstrated promising results, with ORRs of 80% with glofitamab and 72% with mosunetuzumab in RR DLBCL [113,128]. The ORRs observed with this combination appear to be superior to those reported with both glofitamab monotherapy (ORR 58.0%) and mosunetuzumab monotherapy (ORR 42.0%) in RR DLBCL [109,130]. Additionally, results from a phase Ib study demonstrated that glofitamab + Pola-R-CHP (PV, rituximab, cyclophosphamide, doxorubicin, prednisone) in patients with treatment-naive DLBCL demonstrated an ORR of 100% in patients who completed the treatment cycle [121]. These combination regimens have also demonstrated remarkable efficacy in treatment-naive patients in numerous ongoing phase 3 trials comparing their efficacy to standard-of-care treatment [131,132].

Chronic exposure to BiAbs results in continuous T-cell stimulation and subsequent exhaustion, impairing the efficacy of BiAbs/BiTEs [133]. In vitro studies have shown that continuous exposure to BiAbs impairs the T-cell-mediated lysis of neoplastic cells [23]. Exhausted T cells, characterized by the increased expression of inhibitory checkpoint molecules such as PD-1 [134], correlated with disease progression in DCLBL patients receiving glofitamab [135]. Combining BiABs with IMiDs, such as lenalidomide, can enhance T-cell activation via B7-CD28-mediated signaling and reduce T-cell exhaustion and surface PD-1 expression [136]. Phase 1–2 clinical trials combining epcoritamab with lenalidomide + rituximab (R2) in patients with relapsed/refractory FL have demonstrated improved efficacy for the combination regimen than epcoritamab monotherapy (complete response rates of 86% vs. 50%, respectively) [114,137]. Multiple clinical trials evaluating the efficacy of a BiAb and lenalidomide combination for RRFL are underway, with promising phase 1 safety results, but are yet to be conducted in NHL [138,139,140,141]. Another strategy is to activate co-stimulatory receptors, such as CD28 and 4-1BB on T cells, which improve T-cell activation, expansion, and survival [142,143]. Preclinical evidence demonstrated that the antineoplastic capacity of odronextamab was enhanced through its combination with REGN5837, a BiAb that cross-links CD28 on T cells with CD22 on tumor cells. REGN5837 was capable of reactivating exhausted T cells, expanding the intra-tumoral population of T cells, and promoting T-cell persistence, resulting in increased tumor lysis [144]. Additionally, RO7227166, a novel CD19 × 4-1BBL costimulatory BiAb, enhanced the anti-tumor efficacy of glofitamab [145]. Thus, future efforts should be directed towards exploring novel strategies to enhance the T-cell-engaging capacity of BiAbs to dampen the risk of therapeutic resistance facilitated by the immunosuppressive microenvironment of NHL.

## 6. Bispecific T-Cell Engagers and Antibodies in the Treatment of Acute Myelogenous Leukemia

Acute myelogenous leukemia (AML) is characterized by the infiltration of immature leukemic cells in the bone marrow and their accumulation in peripheral blood [146]. Survival rates and outcomes of AML have not improved substantially over the past few decades [146]. Chemotherapy followed by allogeneic stem cell transplantation is the standard of care for AML, but many patients develop relapse or treatment-refractory disease [147]. T-cell-engaging immunotherapies, such as BiAbs, offer a novel approach to target chemotherapy-resistant AML tumor cells. However, the application of BiAbs in AML faces challenges due to the limited target antigens that are ubiquitously expressed on malignant AML cells [148]. Additionally, the overlapping expression of target antigens between malignant AML cells and hematopoietic stem cells raises concerns about on-target off-tumor toxicities, particularly hematologic toxicity and cytopenias.

**Figure 3:**
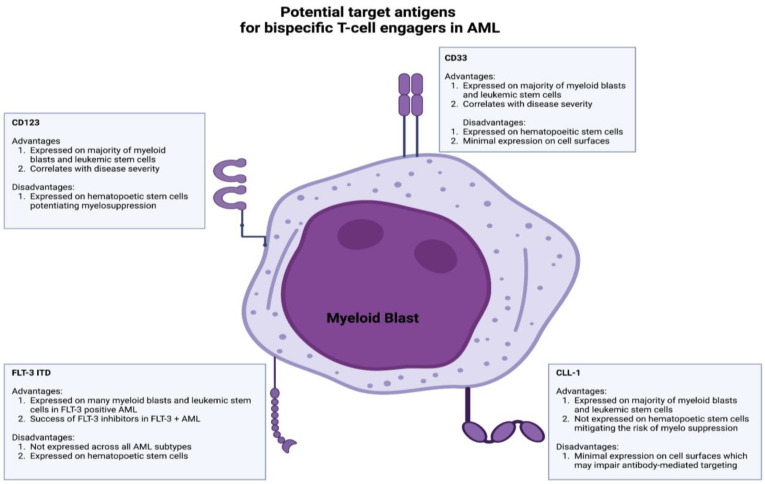
This figure outlines the advantages and disadvantages of the potential target antigens in the development of bispecific antibodies for the treatment of acute myelogenous leukemia. These target antigens, which are expressed on leukemic stem cells and myeloblasts, include CD33, CD123, CLL-1, and FLT-3.

Current bispecific T-cell engagers in AML target many antigens, including CD33, CD123, CLL-1, and FLT-3 (Figure 3) [149]. CD33, a glycoprotein expressed on immature myeloid blasts and leukemic stem cells, has garnered significant interest. CD33 is of particular interest due to its expression on the majority of immature myeloid blasts and leukemic stem cells [149,150]. High CD33 expression correlates with adverse cytogenetic profiles and poor outcomes [151]. However, CD33 is also expressed on hematopoietic stem cells, which increases the risk of myelosuppression [152]. Gemtuzumab ozogamicin, an anti-CD33 antibody–drug conjugate, is approved for relapsed/refractory AML in CD33+ adults [153,154]. Clinical trials evaluating the efficacy of anti-CD33 bispecific T-cell engagers such as AMG 673, AMG-330, and GEM333 have been terminated despite promising preliminary results; however, there are two CD33xCD3 BiAbs in clinical development after the completion of initial phase 1 studies (JNJ-67561244 and AMV564).

CD123, the low-affinity binding subunit of the IL-3 receptor, has emerged as another target antigen for BiTE therapy in AML. CD123 is widely expressed on leukemic stem cells and myeloid blasts and correlates with disease severity and therapy resistance [155,156,157]. Flotetuzumab, a CD123xCD3 dual-affinity retargeting protein (DART), demonstrated anti-leukemic activity with a manageable toxicity profile in relapsed/refractory AML patients [17]. XmAb14045, another CD123xCD3 targeting BiAb, also showed anti-leukemic activity in relapsed/refractory AML patients and is currently being assessed in a phase II clinical trial (NCT05285813) [158]. Other CD123xCD3 BiAbs, such as APVO436 and MGD024, are being evaluated in ongoing phase 1 clinical trials after demonstrating anti-leukemic activity in preclinical studies [159,160].

CLL-1 (CLEC12A) is another potential target antigen for BiTE/BiAb therapy in AML due to its expression on leukemic stem cells and myeloid blasts but not hematopoietic stem cells [161,162,163]. However, CLL-1 has low expression levels on cell surfaces, potentially impairing the antibody activity [162]. MCL-117 is a CLL-1xCD3 bispecific T-cell-engaging antibody that has shown promise in preclinical studies but did not yield optimal clinical responses (NCT03038230) [164,165]. FLT-3, a receptor-type tyrosine kinase, is expressed on leukemic stem cells and myeloid blasts and represents a promising target antigen for BiAbs/BiTE therapy in AML [166,167,168]. FLT-3 inhibitors such as midostaurin and gilteritinib are currently approved for use in FLT-3-mutated AML patients [169,170]. However, FLT-3 expression on hematopoietic stem cells and its limited presence across different AML subtypes pose challenges [171]. CLN-049, a CD3xFLT3 BiTE, demonstrated anti-leukemic activity in preclinical studies and is currently being evaluated in a phase 1 trial (NCT05143996) [172].

BiTEs hold promise in AML treatment, but clinical trials are currently in their infancy (Table 4). There are also several limitations, such as the toxicity of BiAbs/BiTEs, which may hinder the utilization of BiAb/BiTE therapy in AML. Adverse events, such as CRS and cytopenias, are associated with BiTE therapy, but step-up dosing and premedication with steroids have shown to be effective in reducing their severity [17,158]. Strategies to reduce CRS, such as premedication or design modification, should be explored further [173]. Another limitation is potential antigen loss or insufficient expression of the targeted antigen on cell surfaces. For example, single nucleotide polymorphisms (SNP) in the CD33 splicer enhancer region can alter the antibody-binding domain of CD33, resulting in resistance to gemtuzumab ozogamicin [174]. Combining BiAbs targeting multiple antigens on AML cells may overcome therapy resistance due to target antigen loss or structural alterations. The immunosuppressive TME in AML contributes to therapy resistance [175,176,177]. For instance, myeloid-derived suppressor cells (MDSCs) expressing CD14 and CD33 may curb effective anti-tumor responses [178,179]. Additionally, the upregulation of immune checkpoints such as PD-L1 on T cells has been shown to correlate with an impaired anti-tumor T-cell response in AML [180,181,182]. The CD33xCD3 BiAbs AMG330 and AMV564 have demonstrated anti-leukemic activity by activating T cells and modulating MDSCs [183,184,185]. Notably, AMG330 induces potent inflammatory cytokine responses, resulting in the upregulation of PD-L1 on AML cells and subsequent immune evasion [150]. These results indicate that combining BiAbs/BiTEs with immune checkpoint inhibitors such as anti-PD-1 may provide a novel approach to augment bispecific antibody therapy in AML and attenuate treatment resistance.

## 7. Overview of the Toxicities Associated with the Use of Bispecific Antibodies in the Treatment of Hematologic Malignancies

**Figure 4:**
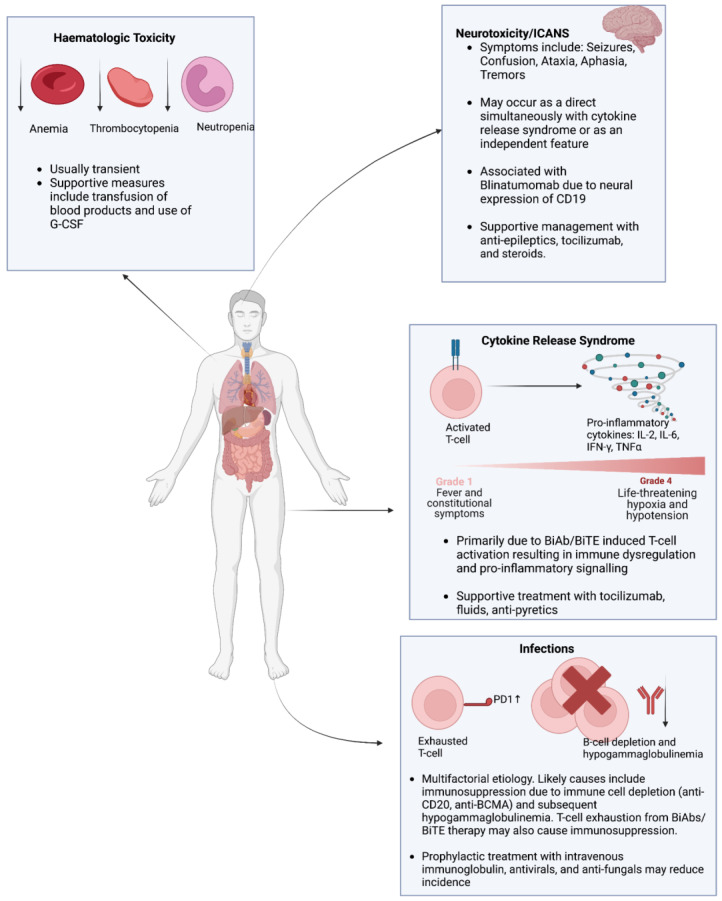
This figure outlines the features of primary toxicities associated with bispecific T-cell engaging therapy in hematological malignancies. These primary toxicities include neurotoxicity/immune effector cell-associated neurotoxicity syndrome, cytokine release syndrome, infections, and hematologic toxicity.

The previous sections have expounded on the therapeutic potential of BiAbs/BiTEs in hematological malignancies. However, ensuring the safety and tolerability of these therapeutic modalities is of paramount importance to incorporate them into treatment protocols for hematological malignancies. The primary adverse events associated with BiAb and BiTE treatment include CRS, infections, hematological toxicity, and neurotoxicity (Figure 4). This section provides an overview of these toxicities associated with BiAbs and BiTE therapy and explores strategies to alleviate these toxicities and minimize their impact.

### 7.1. Cytokine Release Syndrome (CRS)

CRS is characterized by an exaggerated inflammatory response with elevated levels of cytokines interleukin-2 (IL-2), interleukin-6 (IL-6), interferon-gamma (IFN-γ), and tumor necrosis factor-alpha (TNFα) [186,187]. The clinical manifestations of CRS are variable, ranging from mild fever and malaise to severe hypotension and hypoxia [188]. The severity of CRS is graded according to guidelines from the American Society for Transplantation and Cellular Therapy (ASTCT) [189]. Grades 1 and 2 are more common and characterized by non-life-threatening symptoms, whereas grades 3 and 4 require urgent intervention due to the life-threatening nature of symptoms. For instance, a recent meta-analysis of 53 studies found that the rate of CRS in patients treated with BiAbs was 67%, but the rate of severe (grade 3 or 4) CRS was 0.2% [190]. In the context of BiAb and BiTE therapy, CRS occurs due to T-cell activation via the CD3 component of BiTEs/BiAbs [191]. CRS tends to occur primarily during the first cycle of treatment. Additionally, comparing intravenous and subcutaneous administrations of BiAbs/BiTEs, CRS tends to manifest on the first day of intravenous administration compared to the second day of subcutaneous administration [23].

Therefore, intensive monitoring is essential for patients receiving BiAb/BiTE therapy, particularly during the initial 48 hours of dose administration. Step-up dosing has been shown to mitigate the release of inflammatory cytokines and reduce the duration and intensity of CRS [192]. Additionally, the use of BiAbs/BiTEs with a lower affinity for CD3 may ameliorate CRS [192]. Pretreatment with immunosuppressive/immunomodulatory drugs can also attenuate CRS both in vitro and in vivo [193].

When patients develop CRS, immediate supportive care is needed, followed by admission to intensive care units. Supportive care includes the maintenance of normoxia and administration of fluids/antipyretics. Furthermore, the administration of steroids and/or IL-6-blocking mAb tocilizumab can significantly alleviate both the duration and severity of CRS [188].

### 7.2. Infections

It is vital to closely monitor patients receiving BiAb/BiTE therapy for signs of infection. Patients should be screened for opportunistic or reactivation infections such as cytomegalovirus and Epstein–Barr virus [194]. A pooled analysis of 1185 patients receiving BiAb therapy identified a 50% rate of infections, of which 24.5% were graded as severe [195]. The prevalence of hypogammaglobulinemia was reported to be 75.3%. Furthermore, 25.5% of the total deaths were attributed to infections [195]. The incidence of infections in the context of BiAb/BiTE therapy is often multifactorial. Patients with hematological malignancies who receive BiAb/BiTE therapy often have refractory disease with prior exposure to multiple lines of therapy, increasing their risk of infection. Moreover, patients with active hematological malignancies are often neutropenic due to impaired hematopoiesis. Anti-BCMA BiAb/BiTEs in multiple myeloma and anti-CD20 BiAb/BiTEs in non-Hodgkin’s lymphoma impair B-cell function, resulting in profound hypogammaglobulinemia [196,197]. Lastly, continuous T-cell stimulation by BiAbs/BiTEs may promote T-cell exhaustion, increasing the susceptibility to infections [24]. Preventative strategies to minimize the infection risk in BiAb/BiTE therapy include prophylactic IVIG and antimicrobials (antibiotics, antivirals, and antifungals) [194,195,196].

### 7.3. Hematologic Toxicity

Patients treated with BiAbs/BiTEs are predisposed to the development of hematological toxicities, including anemia, thrombocytopenia, and neutropenia. The exact mechanism behind the development of these toxicities remains unknown but may be linked to therapy-induced pro-inflammatory cytokine release and/or the impairment of hematopoiesis [198,199]. Supportive measures, such as the transfusion of blood products and granulocyte colony-stimulating factors, should be considered to improve hematologic parameters and reduce the infection risk.

### 7.4. Neurotoxicity

Neurotoxicity in the context of BiAb/BiTE therapy can arise either from CRS or as a consequence of immune effector cell-associated neurotoxicity syndrome (ICANS) [200]. The symptoms of neurotoxicity due to CRS or ICANS are variable and may include seizures, confusion, tremors, dysphasia/aphasia, and ataxia [200]. The severity of ICANS is graded based on the ASTCT guidelines [189]. Strategies to alleviate the risk of ICANS are similar to those employed in CRS, including steroids and tocilizumab. Notably, blinatumomab carriers the highest risk of neurotoxicity, likely due to CD19 co-expression in neural tissue, necessitating a high index of suspicion for ICANS for patients receiving blinatumomab [71,125].

## 8. Conclusions and Future Directions

BiAbs/BiTEs have transformed the treatment landscape for advanced hematological malignancies, with several approved BiAbs/BiTEs showing promising efficacy and favorable safety profiles. Additionally, early-stage clinical trials of numerous other BiTEs have demonstrated encouraging anti-neoplastic activity, raising optimism for their approval in the coming years.

However, the efficacy and tolerability of certain BiAbs/BiTEs warrant further exploration. Antigen escape is a major resistance mechanism to BiAb/BiTE therapy. Elucidating additional tumor-associated target antigens and exploring combinatorial, multi-antigenic BiAb/BiTE may counteract antigen escape. Additionally, the immunosuppressive TME in hematological malignancies is a significant contributor to BiAb/BiTE resistance. Investigating strategies to normalize the tumor microenvironment, such as immune checkpoint inhibitors and immunomodulatory agents, may enhance the efficacy of BiAbs/BiTEs and mitigate therapeutic resistance.

Presently, much of the BiAb/BiTE research in hematological malignancies focuses on patients with relapsed/refractory disease, but it is plausible that patients in the early stages of their disease may respond to BiAbs/BiTEs more favorably due to their lower tumor burden and a TME more conducive to anti-tumor immune responses. We have already discussed how T-cell dysfunction consequent to exposure to multiple lines of cancer therapy has been associated with resistance to BiABs, early-stage disease being associated with a better therapeutic response to BiTEs, and clinical trials being underway to assess the efficacy of BiAbs/BiTEs in early-stage hematologic malignancies. Exploring novel BiAb engineering strategies such as trispecific antibodies—which target more tumor antigens and minimize the risk of antigen escape—is also important. Incorporating natural killer cell engagers may also synergize with bispecific T-cell engagers and potentially enhance the anti-tumor immune response.

In conclusion, the field of bispecific T-cell engagers holds tremendous therapeutic potential, and we eagerly anticipate further progress from future preclinical studies and clinical trials. The continued advancement of these therapies is expected to have a significant impact on the treatment of hematological malignancies, bringing hope to patients and healthcare professionals alike.

## Figures and Tables

**Table 1:** Phase 1 and 2 studies evaluating the safety and efficacy of bispecific antibodies targeting BCMA, GPRC5D, and FcRH5 in multiple myeloma.

BiAB, Trial	Targets	BiAB Structure	N	Design	ORR, CR (%)	CRS (All Grade, ≥Grade 3) %	ICANS (%)	Infections (%)
Teclistamab (Ph1-2, NCT04557098) [32]	BCMAxCD3	Humanized IgG Fc	165	SQ, weekly injection at dose of 1.5 mg/kg. Step-up doses of 0.06 mg and 0.3 mg per kilogram.	63.0, 39.4	72.1, 0.6	3.0	76.4
Elranatamab (Ph2, NCT04649359) [33]	BCMAxCD3	Humanized IgG2a	123	SQ, weekly injection at a dose of 76 mg for a 28-day cycle. Two step-up doses at 12 mg and 32 mg.	61.0, 27.6	56.3, 0.0	3.4	61.8
Linvoseltamab (Ph2, NCT03761108) [34]	BCMAxCD3	Fc Fab arms	252	Two cohorts received doses of 50 mg and 200 mg, respectively. IV, with two step-up doses. A protocol amendment allowed pts who progressed at 50 mg to dose escalate to 200 mg.	50 mg cohort: 50.0, 20.2 200 mg cohort: 64.0, 24.1	50 mg cohort: 53.0, 1.0 200 mg cohort: 37.0, 2.0	Grade 3 or 4 50 mg cohort: 1.0 200 mg cohort: 2.0	50 mg cohort: 59.0 200 mg cohort: 43.0
Abbv-383 (Ph1, NCT03933735) [35]	BCMAxCD3	IgG4 Fc	124	IV, once every 3 weeks. Doses of 40 mg and 60 mg for escalation and expansion cohorts.	57.0, 29.0	40 mg cohort: 83.0, 0.0 60 mg cohort: 72.0, 2.0	NR	40 mg cohort: 50.0 60 mg cohort: 43.0
Talquetamab (Ph1, NCT03399799) [36]	GPRC5DxCD3	Humanized IgG4	232	102 patients IV weekly or every other week at doses from 0.5 to 180 μg per kilogram of body weight. 130 patients SQ weekly, every other week, or monthly at doses from 5 to 1600 μg per kilogram.	At SQ doses of 405 μg/kg: 70.0, 23.0 and 800 μg/kg: 64.0, 23.0	At SQ doses of 405 μg/kg: 77.0, 3.0 and 800 μg/kg: 80.0, 0.0 At IV doses: 49.0, 5.0	NR	NR
Cevostamab (Ph1, NCT03275103) [41]	FcRH5xCD3	Humanized IgG1	160	IV administration in 21-day cycles. Two step-up doses.	At 160 mg dose: 54% At 90 mg dose: 36.7	80.0, 1.3	NR	42.5, 18.8

BiAb = bispecific antibody. CRS = cytokine release syndrome. ICANS = immune effector cell-associated neurotoxicity syndrome. ORR = overall response rate. CR = complete response. NR = not reported. SQ = subcutaneous. IV = intravenous.

**Table 2:** Selected clinical trials evaluating the efficacy of blinatumomab in different patient populations.

First Author, Year	Phase	N	Study Design and Patient Population	Outcomes	Adverse Events
Kantarjian et al. 2017 [60]	3	405	Heavily pretreated relapsed/refractory Philadelphia-negative B-ALL. Randomized 2:1 comparison between blinatumomab and standard-of-care chemotherapy.	Median overall survival in blinatumomab group 7.7 months vs. 4.0 months in standard-of-care group. Complete hematologic remission in 34% in the blinatumomab group vs. 16% in the standard-of-care group.	Infection in 34.1% of the blinatumomab group vs. 52.3% in the standard-of-care group. Neurotoxicity in 9.4% in the blinatumomab group vs. 8.3% in the standard-of-care group.
Gökbuget et al. 2018 [67]	2	116	Open-label, single-arm study, adults with B-cell precursor ALL in hematologic complete remission with MRD (≥10^−3^).	MRD clearance in 78% of patients. Relapse-free survival at 18 months 54%. Median overall survival of 36.5 months.	Cytokine release syndrome in 3%. Neurotoxicity grade 3 in 10%, grade 4 in 3%.
Brown et al. 2021 [69]	3	208	Ages 1–30 years with first relapse B-ALL. Randomized between 2 cycles of blinatumomab and 2 cycles multi-agent chemotherapy.	2-year disease free survival 54.4% in the blinatumomab group vs. 39.0% in the chemotherapy group. 2-year overall survival 71.3% in the blinatumomab group vs. 58.4% in the chemotherapy group.	Infection in 15.0% of the blinatumomab group vs. 65.0% in the chemotherapy group.
Litzow et al. 2022 [73]	3	224	Patients with negative MRD (<0.01%) post-induction therapy were randomized to either receive conventional consolidation chemotherapy or blinatumomab in addition to conventional consolidation.	Upper boundary for efficacy analysis was crossed in favor of blinatumomab, with a significant improvement in overall survival in favor of blinatumomab arm. Median overall survival not reached vs. 71.4 months, hazard ratio 0.42, *p* = 0.003.	NR
Salek et al. 2022 [74]	2	29	Single cycle of blinatumomab followed by high-dose chemotherapy in induction therapy for Philadelphia-negative adult ALL.	93% of patients achieved complete hematological remission after induction, of which 52% were complete molecular remissions.	Febrile neutropenia in 15%, and hepatotoxicity in 11%. No neurotoxicity observed.
Foà et al. 2020 [15]	2	63	Philadelphia-positive ALL patients. Single-arm trial in which Dasatinib plus glucocorticoids were administered, followed by two cycles of blinatumomab.	Complete remission achieved in 98%. At median follow-up of 18 months, overall survival was 95% with disease-free survival of 88%.	Grade ≥ 3 adverse events included cytomegalovirus reactivation in 6 patients, neutropenia in 4 patients, and neurotoxicity in one patient.

**Table 4:** Past and present clinical trials evaluating the use of bispecific T-cell engagers and antibodies in acute myelogenous leukemia.

Trial ID	Antibody Name	Targets	Patient Population	Phase	Primary Outcomes	Status
NCT02520427	AMG 330	CD33xCD3	Relapsed/refractory AML, MDS	1	Safety	Terminated
NCT03224819	AMG 673	CD33xCD3	Relapsed/refractory AML	1	Safety	Terminated
NCT03516760	GEM333	CD33xCD3	Relapsed/refractory AML	1	Safety	Terminated
NCT03915379	JNJ-67571244	CD33xCD3	Relapsed/refractory AML, MDS	1	Safety and efficacy	Completed
NCT03144245	AMV564	CD33xCD3	Relapsed/refractory AML	1	Safety and efficacy	Completed
NCT04582864	Flotetuzumab	CD123xCD3	Relapsed/refractory AML	2	Efficacy	Recruiting
NCT05285813	XmAb14045	CD123xCD3	Relapsed/refractory AML, MDS	2	Efficacy	Recruiting
NCT03647800	APVO436	CD123xCD3	Relapsed/refractory AML, MDS	1	Safety	Recruiting
NCT05362773	MGD024	CD123xCD3	Relapsed/refractory AML, MDS, Hodgkin’s lymphoma, B-cell leukemia, hairy cell leukemia, CML, systemic mastocytosis	1	Safety	Recruiting
NCT02715011	JNJ-63709178	CD123xCD3	Relapsed/refractory AML	1	Safety	Completed
NCT03038230	MCLA-117	CLL-1xCD3	Relapsed/refractory AML	1	Safety	Halted
NCT05143996	CLN-049	FLT-3xCD3	Relapsed/refractory AML, MDS	1	Safety	Recruiting

## References

[1] Maude S.L., Frey N., Shaw P.A., Aplenc R., Barrett D.M., Bunin N.J., Chew A., Gonzalez V.E., Zheng Z., Lacey S.F. (2014). Chimeric antigen receptor T cells for sustained remissions in leukemia. N. Engl. J. Med..

[2] Kochenderfer J.N., Dudley M.E., Kassim S.H., Somerville R.P., Carpenter R.O., Stetler-Stevenson M., Yang J.C., Phan G.Q., Hughes M.S., Sherry R.M. (2015). Chemotherapy-refractory diffuse large B-cell lymphoma and indolent B-cell malignancies can be effectively treated with autologous T cells expressing an anti-CD19 chimeric antigen receptor. J. Clin. Oncol..

[3] Munshi N.C., Anderson L.D., Shah N., Madduri D., Berdeja J., Lonial S., Raje N., Lin Y., Siegel D., Oriol A. (2021). Idecabtagene Vicleucel in Relapsed and Refractory Multiple Myeloma. N. Engl. J. Med..

[4] June C.H., Sadelain M. (2018). Chimeric Antigen Receptor Therapy. N. Engl. J. Med..

[5] Tully S., Feng Z., Grindrod K., McFarlane T., Chan K.K.W., Wong W.W.L. (2019). Impact of Increasing Wait Times on Overall Mortality of Chimeric Antigen Receptor T-Cell Therapy in Large B-Cell Lymphoma: A Discrete Event Simulation Model. JCO Clin. Cancer Inform..

[6] Sterner R.C., Sterner R.M. (2021). CAR-T cell therapy: Current limitations and potential strategies. Blood Cancer J..

[7] Subklewe M. (2021). BiTEs better than CAR T cells. Blood Adv..

[8] Topp M.S., Kufer P., Gökbuget N., Goebeler M., Klinger M., Neumann S., Horst H.A., Raff T., Viardot A., Schmid M. (2011). Targeted therapy with the T-cell-engaging antibody blinatumomab of chemotherapy-refractory minimal residual disease in B-lineage acute lymphoblastic leukemia patients results in high response rate and prolonged leukemia-free survival. J. Clin. Oncol..

[9] Allen C., Zeidan A.M., Bewersdorf J.P. (2021). BiTEs, DARTS, BiKEs and TriKEs—Are Antibody Based Therapies Changing the Future Treatment of AML?. Life.

[10] Labrijn A.F., Janmaat M.L., Reichert J.M., Parren P.W.H.I. (2019). Bispecific antibodies: A mechanistic review of the pipeline. Nat. Rev. Drug Discov..

[11] Wang Q., Chen Y., Park J., Liu X., Hu Y., Wang T., McFarland K., Betenbaugh M.J. (2019). Design and Production of Bispecific Antibodies. Antibodies.

[12] Thakur A., Huang M., Lum L.G. (2018). Bispecific antibody based therapeutics: Strengths and challenges. Blood Rev..

[13] Belmontes B., Sawant D.V., Zhong W., Tan H., Kaul A., Aeffner F., O’Brien S.A., Chun M., Noubade R., Eng J. (2021). Immunotherapy combinations overcome resistance to bispecific T cell engager treatment in T cell–cold solid tumors. Sci. Transl. Med..

[14] Zhao L., Li S., Wei X., Qi X., Liu D., Liu L., Wen F., Zhang J.S., Wang F., Liu Z.L. (2022). A novel CD19/CD22/CD3 trispecific antibody enhances therapeutic efficacy and overcomes immune escape against B-ALL. Blood.

[15] Foà R., Bassan R., Vitale A., Elia L., Piciocchi A., Puzzolo M.C., Canichella M., Viero P., Ferrara F., Lunghi M. (2020). Dasatinib-Blinatumomab for Ph-Positive Acute Lymphoblastic Leukemia in Adults. N. Engl. J. Med..

[16] Bröske A.E., Korfi K., Belousov A., Wilson S., Ooi C.H., Bolen C.R., Canamero M., Alcaide E.G., James I., Piccione E.C. (2022). Pharmacodynamics and molecular correlates of response to glofitamab in relapsed/refractory non-Hodgkin lymphoma. Blood Adv..

[17] Uy G.L., Aldoss I., Foster M.C., Sayre P.H., Wieduwilt M.J., Advani A.S., Godwin J.E., Arellano M.L., Sweet K.L., Emadi A. (2021). Flotetuzumab as salvage immunotherapy for refractory acute myeloid leukemia. Blood.

[18] Arenas E.J., Martínez-Sabadell A., Rius Ruiz I., Román Alonso M., Escorihuela M., Luque A., Fajardo C.A., Gros A., Klein C., Arribas J. (2021). Acquired cancer cell resistance to T cell bispecific antibodies and CAR T targeting HER2 through JAK2 down-modulation. Nat. Commun..

[19] Verkleij C.P.M., Broekmans M.E.C., van Duin M., Frerichs K.A., Kuiper R., de Jonge A.V., Kaiser M., Morgan G., Axel A., Boominathan R. (2021). Preclinical activity and determinants of response of the GPRC5DxCD3 bispecific antibody talquetamab in multiple myeloma. Blood Adv..

[20] van de Donk N.W., Bahlis N., Mateos M.V., Weisel K., Dholaria B., Garfall A.L., Goldschmidt H., Martin T.G., Morillo D., Reece D.E. (2022). S183: Novel Combination Immunotherapy for the Treatment of Relapsed/Refractory Multiple Myeloma: Updated Phase 1B Results for Talquetamab (A GPRC5D X CD3 Bispecific Antibody) in Combination with Daratumumab. HemaSphere.

[21] Duell J., Dittrich M., Bedke T., Mueller T., Eisele F., Rosenwald A., Rasche L., Hartmann E., Dandekar T., Einsele H. (2017). Frequency of regulatory T cells determines the outcome of the T-cell-engaging antibody blinatumomab in patients with B-precursor ALL. Leukemia.

[22] Cortes-Selva D., Casneuf T., Vishwamitra D., Stein S., Perova T., Skerget S., Ramos E., van Steenbergen L., De Maeyer D., Boominathan R. (2022). Teclistamab, a B-Cell Maturation Antigen (BCMA) x CD3 Bispecific Antibody, in Patients with Relapsed/Refractory Multiple Myeloma (RRMM): Correlative Analyses from MajesTEC-1. Blood.

[23] van de Donk N., Zweegman S. (2023). T-cell-engaging bispecific antibodies in cancer. Lancet.

[24] Philipp N., Kazerani M., Nicholls A., Vick B., Wulf J., Straub T., Scheurer M., Muth A., Hänel G., Nixdorf D. (2022). T-cell exhaustion induced by continuous bispecific molecule exposure is ameliorated by treatment-free intervals. Blood.

[25] Meermeier E.W., Welsh S.J., Sharik M.E., Du M.T., Garbitt V.M., Riggs D.L., Shi C.X., Stein C.K., Bergsagel M., Chau B. (2021). Tumor burden limits bispecific antibody efficacy through T cell exhaustion averted by concurrent cytotoxic therapy. Blood Cancer Discov..

[26] Palumbo A., Avet-Loiseau H., Oliva S., Lokhorst H.M., Goldschmidt H., Rosinol L., Richardson P., Caltagirone S., Lahuerta J.J., Facon T. (2015). Revised International Staging System for Multiple Myeloma: A Report from International Myeloma Working Group. J. Clin. Oncol..

[27] Marcon C., Simeon V., Deias P., Facchin G., Corso A., Derudas D., Montefusco V., Offidani M., Petrucci M.T., Zambello R. (2022). Experts’ consensus on the definition and management of high risk multiple myeloma. Front. Oncol..

[28] Mateos M.V., Weisel K., De Stefano V., Goldschmidt H., Delforge M., Mohty M., Cavo M., Vij R., Lindsey-Hill J., Dytfeld D. (2022). LocoMMotion: A prospective, non-interventional, multinational study of real-life current standards of care in patients with relapsed and/or refractory multiple myeloma. Leukemia.

[29] Shah N., Chari A., Scott E., Mezzi K., Usmani S.Z. (2020). B-cell maturation antigen (BCMA) in multiple myeloma: Rationale for targeting and current therapeutic approaches. Leukemia.

[30] Ghermezi M., Li M., Vardanyan S., Harutyunyan N.M., Gottlieb J., Berenson A., Spektor T.M., Andreu-Vieyra C., Petraki S., Sanchez E. (2017). Serum B-cell maturation antigen: A novel biomarker to predict outcomes for multiple myeloma patients. Haematologica.

[31] Swan D., Murphy P., Glavey S., Quinn J. (2023). Bispecific Antibodies in Multiple Myeloma: Opportunities to Enhance Efficacy and Improve Safety. Cancers.

[32] Moreau P., Garfall A.L., van de Donk N., Nahi H., San-Miguel J.F., Oriol A., Nooka A.K., Martin T., Rosinol L., Chari A. (2022). Teclistamab in Relapsed or Refractory Multiple Myeloma. N. Engl. J. Med..

[33] Bahlis N.J., Tomasson M.H., Mohty M., Niesvizky R., Nooka A.K., Manier S., Maisel C., Jethava Y., Martinez-Lopez J., Prince H.M. (2022). Efficacy and Safety of Elranatamab in Patients with Relapsed/Refractory Multiple Myeloma Naïve to B-Cell Maturation Antigen (BCMA)-Directed Therapies: Results from Cohort a of the Magnetismm-3 Study. Blood.

[34] Lee H.C., Bumma N., Richter J.R., Dhodapkar M.V., Hoffman J.E., Suvannasankha A., Zonder J.A., Shah M.R., Lentzsch S., Maly J.J. (2023). LINKER-MM1 study: Linvoseltamab (REGN5458) in patients with relapsed/refractory multiple myeloma. J. Clin. Oncol..

[35] D’Souza A., Shah N., Rodriguez C., Voorhees P.M., Weisel K., Bueno O.F., Pothacamury R.K., Freise K.J., Yue S., Ross J.A. (2022). A Phase I First-in-Human Study of ABBV-383, a B-Cell Maturation Antigen × CD3 Bispecific T-Cell Redirecting Antibody, in Patients with Relapsed/Refractory Multiple Myeloma. J. Clin. Oncol..

[36] Truger M.S., Duell J., Zhou X., Heimeshoff L., Ruckdeschel A., John M., Riedel A., Hüper S., Peter J., Walter W. (2021). Single- and double-hit events in genes encoding immune targets before and after T cell-engaging antibody therapy in MM. Blood Adv..

[37] Nath K., Costa B.A., Mailankody S. (2023). GPRC5D as a novel immunotherapeutic target in multiple myeloma. Nat. Rev. Clin. Oncol..

[38] Chari A., Minnema M.C., Berdeja J.G., Oriol A., van de Donk N., Rodríguez-Otero P., Askari E., Mateos M.V., Costa L.J., Caers J. (2022). Talquetamab, a T-Cell-Redirecting GPRC5D Bispecific Antibody for Multiple Myeloma. N. Engl. J. Med..

[39] Carlo-Stella C., Mazza R., Manier S., Facon T., Yoon S.-S., Koh Y., Harrison S.J., Er J., Pinto A., Volzone F. (2022). RG6234, a GPRC5DxCD3 T-Cell Engaging Bispecific Antibody, Is Highly Active in Patients (pts) with Relapsed/Refractory Multiple Myeloma (RRMM): Updated Intravenous (IV) and First Subcutaneous (SC) Results from a Phase I Dose-Escalation Study. Blood.

[40] Li J., Stagg N.J., Johnston J., Harris M.J., Menzies S.A., DiCara D., Clark V., Hristopoulos M., Cook R., Slaga D. (2017). Membrane-Proximal Epitope Facilitates Efficient T Cell Synapse Formation by Anti-FcRH5/CD3 and Is a Requirement for Myeloma Cell Killing. Cancer Cell.

[41] Trudel S., Cohen A.D., Krishnan A.Y., Fonseca R., Spencer A., Berdeja J.G., Lesokhin A., Forsberg P.A., Laubach J.P., Costa L.J. (2021). Cevostamab Monotherapy Continues to Show Clinically Meaningful Activity and Manageable Safety in Patients with Heavily Pre-Treated Relapsed/Refractory Multiple Myeloma (RRMM): Updated Results from an Ongoing Phase I Study. Blood.

[42] van de Donk N., Richardson P.G., Malavasi F. (2018). CD38 antibodies in multiple myeloma: Back to the future. Blood.

[43] Mohan S.R., Costa Chase C., Berdeja J.G., Karlin L., Belhadj K., Perrot A., Moreau P., Touzeau C., Chalopin T., Lesokhin A.M. (2022). Initial Results of Dose Escalation of ISB 1342, a Novel CD3xCD38 Bispecific Antibody, in Patients with Relapsed/Refractory Multiple Myeloma (RRMM). Blood.

[44] Akhmetzyanova I., McCarron M.J., Parekh S., Chesi M., Bergsagel P.L., Fooksman D.R. (2020). Dynamic CD138 surface expression regulates switch between myeloma growth and dissemination. Leukemia.

[45] Morillo D., Gatt M.E., Sebag M., Kim K., Min C.-K., Oriol A., Ocio E.M., Yoon S.-S., Mateos M.-V., Chu M. (2023). First results from the RedirecTT-1 study with teclistamab (tec) + talquetamab (tal) simultaneously targeting BCMA and GPRC5D in patients (pts) with relapsed/refractory multiple myeloma (RRMM). J. Clin. Oncol..

[46] Shearer T., Williams R.L., Johnson M., Cendrowicz E., Leonowens C., Smith M., Baughman T., Breitbach C.J., Smith L.M., Burgess M. (2022). Pharmacodynamic Effects of Nirogacestat, a Gamma Secretase Inhibitor, on B-Cell Maturation Antigen in Healthy Participants. Blood.

[47] Uckun F.M. (2021). Overcoming the Immunosuppressive Tumor Microenvironment in Multiple Myeloma. Cancers.

[48] Visram A., Dasari S., Anderson E., Kumar S., Kourelis T.V. (2021). Relapsed multiple myeloma demonstrates distinct patterns of immune microenvironment and malignant cell-mediated immunosuppression. Blood Cancer J..

[49] Feyler S., Scott G.B., Parrish C., Jarmin S., Evans P., Short M., McKinley K., Selby P.J., Cook G. (2012). Tumour cell generation of inducible regulatory T-cells in multiple myeloma is contact-dependent and antigen-presenting cell-independent. PLoS ONE.

[50] Soekojo C.Y., Chng W.J. (2022). The evolution of immune dysfunction in multiple myeloma. Eur. J. Haematol..

[51] Krämer I., Engelhardt M., Fichtner S., Neuber B., Medenhoff S., Bertsch U., Hillengass J., Raab M.S., Hose D., Ho A.D. (2016). Lenalidomide enhances myeloma-specific T-cell responses in vivo and in vitro. Oncoimmunology.

[52] Krejcik J., Casneuf T., Nijhof I.S., Verbist B., Bald J., Plesner T., Syed K., Liu K., van de Donk N.W., Weiss B.M. (2016). Daratumumab depletes CD38+ immune regulatory cells, promotes T-cell expansion, and skews T-cell repertoire in multiple myeloma. Blood.

[53] Searle E., Quach H., Wong S.W., Costa L.J., Hulin C., Janowski W., Berdeja J., Anguille S., Matous J.V., Touzeau C. (2022). Teclistamab in Combination with Subcutaneous Daratumumab and Lenalidomide in Patients with Multiple Myeloma: Results from One Cohort of MajesTEC-2, a Phase1b, Multicohort Study. Blood.

[54] Rodríguez-Otero P., D’Souza A., Reece D.E., van de Donk N.W.C.J., Chari A., Krishnan A.Y., Martin T.G., Mateos M.-V., Morillo D., Hurd D.D. (2022). A novel, immunotherapy-based approach for the treatment of relapsed/refractory multiple myeloma (RRMM): Updated phase 1b results for daratumumab in combination with teclistamab (a BCMA x CD3 bispecific antibody). J. Clin. Oncol..

[55] Rosenblatt J., Avigan D. (2017). Targeting the PD-1/PD-L1 axis in multiple myeloma: A dream or a reality?. Blood.

[56] Duffield A.S., Mullighan C.G., Borowitz M.J. (2023). International Consensus Classification of acute lymphoblastic leukemia/lymphoma. Virchows Arch..

[57] Terwilliger T., Abdul-Hay M. (2017). Acute lymphoblastic leukemia: A comprehensive review and 2017 update. Blood Cancer J..

[58] DuVall A.S., Sheade J., Anderson D., Yates S.J., Stock W. (2022). Updates in the Management of Relapsed and Refractory Acute Lymphoblastic Leukemia: An Urgent Plea for New Treatments Is Being Answered!. JCO Oncol. Pract..

[59] Wang K., Wei G., Liu D. (2012). CD19: A biomarker for B cell development, lymphoma diagnosis and therapy. Exp. Hematol. Oncol..

[60] Kantarjian H., Stein A., Gökbuget N., Fielding A.K., Schuh A.C., Ribera J.M., Wei A., Dombret H., Foà R., Bassan R. (2017). Blinatumomab versus Chemotherapy for Advanced Acute Lymphoblastic Leukemia. N. Engl. J. Med..

[61] Bassan R., Spinelli O., Oldani E., Intermesoli T., Tosi M., Peruta B., Rossi G., Borlenghi E., Pogliani E.M., Terruzzi E. (2009). Improved risk classification for risk-specific therapy based on the molecular study of minimal residual disease (MRD) in adult acute lymphoblastic leukemia (ALL). Blood.

[62] Brüggemann M., Raff T., Flohr T., Gökbuget N., Nakao M., Droese J., Lüschen S., Pott C., Ritgen M., Scheuring U. (2006). Clinical significance of minimal residual disease quantification in adult patients with standard-risk acute lymphoblastic leukemia. Blood.

[63] Gökbuget N., Kneba M., Raff T., Trautmann H., Bartram C.R., Arnold R., Fietkau R., Freund M., Ganser A., Ludwig W.D. (2012). Adult patients with acute lymphoblastic leukemia and molecular failure display a poor prognosis and are candidates for stem cell transplantation and targeted therapies. Blood.

[64] Raff T., Gökbuget N., Lüschen S., Reutzel R., Ritgen M., Irmer S., Böttcher S., Horst H.A., Kneba M., Hoelzer D. (2007). Molecular relapse in adult standard-risk ALL patients detected by prospective MRD monitoring during and after maintenance treatment: Data from the GMALL 06/99 and 07/03 trials. Blood.

[65] Borowitz M.J., Wood B.L., Devidas M., Loh M.L., Raetz E.A., Salzer W.L., Nachman J.B., Carroll A.J., Heerema N.A., Gastier-Foster J.M. (2015). Prognostic significance of minimal residual disease in high risk B-ALL: A report from Children’s Oncology Group study AALL0232. Blood.

[66] Berry D.A., Zhou S., Higley H., Mukundan L., Fu S., Reaman G.H., Wood B.L., Kelloff G.J., Jessup J.M., Radich J.P. (2017). Association of Minimal Residual Disease with Clinical Outcome in Pediatric and Adult Acute Lymphoblastic Leukemia: A Meta-analysis. JAMA Oncol..

[67] Gökbuget N., Dombret H., Bonifacio M., Reichle A., Graux C., Faul C., Diedrich H., Topp M.S., Brüggemann M., Horst H.A. (2018). Blinatumomab for minimal residual disease in adults with B-cell precursor acute lymphoblastic leukemia. Blood.

[68] Locatelli F., Zugmaier G., Rizzari C., Morris J.D., Gruhn B., Klingebiel T., Parasole R., Linderkamp C., Flotho C., Petit A. (2021). Effect of Blinatumomab vs Chemotherapy on Event-Free Survival Among Children with High-risk First-Relapse B-Cell Acute Lymphoblastic Leukemia: A Randomized Clinical Trial. Jama.

[69] Brown P.A., Ji L., Xu X., Devidas M., Hogan L.E., Borowitz M.J., Raetz E.A., Zugmaier G., Sharon E., Bernhardt M.B. (2021). Effect of Postreinduction Therapy Consolidation with Blinatumomab vs Chemotherapy on Disease-Free Survival in Children, Adolescents, and Young Adults with First Relapse of B-Cell Acute Lymphoblastic Leukemia: A Randomized Clinical Trial. Jama.

[70] Topp M.S., Gökbuget N., Stein A.S., Zugmaier G., O’Brien S., Bargou R.C., Dombret H., Fielding A.K., Heffner L., Larson R.A. (2015). Safety and activity of blinatumomab for adult patients with relapsed or refractory B-precursor acute lymphoblastic leukaemia: A multicentre, single-arm, phase 2 study. Lancet Oncol..

[71] Stein A.S., Schiller G., Benjamin R., Jia C., Zhang A., Zhu M., Zimmerman Z., Topp M.S. (2019). Neurologic adverse events in patients with relapsed/refractory acute lymphoblastic leukemia treated with blinatumomab: Management and mitigating factors. Ann. Hematol..

[72] Jen E.Y., Xu Q., Schetter A., Przepiorka D., Shen Y.L., Roscoe D., Sridhara R., Deisseroth A., Philip R., Farrell A.T. (2019). FDA Approval: Blinatumomab for Patients with B-cell Precursor Acute Lymphoblastic Leukemia in Morphologic Remission with Minimal Residual Disease. Clin. Cancer Res..

[73] Litzow M.R., Sun Z., Paietta E., Mattison R.J., Lazarus H.M., Rowe J.M., Arber D.A., Mullighan C.G., Willman C.L., Zhang Y. (2022). Consolidation Therapy with Blinatumomab Improves Overall Survival in Newly Diagnosed Adult Patients with B-Lineage Acute Lymphoblastic Leukemia in Measurable Residual Disease Negative Remission: Results from the ECOG-ACRIN E1910 Randomized Phase III National Cooperative Clinical Trials Network Trial. Blood.

[74] Salek C., Folber F., Hrabovsky S., Koristek Z., Horacek J.M., Fronkova E., Soukup P., Benkova K., Cetkovsky P., Trka J. (2022). Single Cycle of Blinatumomab Followed By High-Dose Chemotherapy in the Induction Therapy for Ph-Negative Acute Lymphoblastic Leukemia in Adults. Primary Endpoint Analysis of the Blina-Cell Trial. Blood.

[75] Foà R., Chiaretti S. (2022). Philadelphia Chromosome-Positive Acute Lymphoblastic Leukemia. N. Engl. J. Med..

[76] Jabbour E., Haddad F.G., Short N.J., Kantarjian H. (2022). Treatment of Adults with Philadelphia Chromosome-Positive Acute Lymphoblastic Leukemia-from Intensive Chemotherapy Combinations to Chemotherapy-Free Regimens: A Review. JAMA Oncol..

[77] Jabbour E., Short N.J., Jain N., Huang X., Montalban-Bravo G., Banerjee P., Rezvani K., Jiang X., Kim K.H., Kanagal-Shamanna R. (2023). Ponatinib and blinatumomab for Philadelphia chromosome-positive acute lymphoblastic leukaemia: A US, single-centre, single-arm, phase 2 trial. Lancet Haematol..

[78] Topp M.S., Gökbuget N., Zugmaier G., Degenhard E., Goebeler M.E., Klinger M., Neumann S.A., Horst H.A., Raff T., Viardot A. (2012). Long-term follow-up of hematologic relapse-free survival in a phase 2 study of blinatumomab in patients with MRD in B-lineage ALL. Blood.

[79] Lussana F., Gritti G., Rambaldi A. (2021). Immunotherapy of Acute Lymphoblastic Leukemia and Lymphoma with T Cell-Redirected Bispecific Antibodies. J. Clin. Oncol..

[80] Zhao Y., Aldoss I., Qu C., Crawford J.C., Gu Z., Allen E.K., Zamora A.E., Alexander T.B., Wang J., Goto H. (2021). Tumor-intrinsic and -extrinsic determinants of response to blinatumomab in adults with B-ALL. Blood.

[81] Braig F., Brandt A., Goebeler M., Tony H.P., Kurze A.K., Nollau P., Bumm T., Böttcher S., Bargou R.C., Binder M. (2017). Resistance to anti-CD19/CD3 BiTE in acute lymphoblastic leukemia may be mediated by disrupted CD19 membrane trafficking. Blood.

[82] (2022). Modulation of CD19 surface expression in B cell acute lymphoblastic leukemia. Nat. Immunol..

[83] Lanza F., Maffini E., Rondoni M., Massari E., Faini A.C., Malavasi F. (2020). CD22 Expression in B-Cell Acute Lymphoblastic Leukemia: Biological Significance and Implications for Inotuzumab Therapy in Adults. Cancers.

[84] Spiegel J.Y., Patel S., Muffly L., Hossain N.M., Oak J., Baird J.H., Frank M.J., Shiraz P., Sahaf B., Craig J. (2021). CAR T cells with dual targeting of CD19 and CD22 in adult patients with recurrent or refractory B cell malignancies: A phase 1 trial. Nat. Med..

[85] Roddie C., Lekakis L.J., Marzolini M.A.V., Ramakrishnan A., Zhang Y., Hu Y., Peddareddigari V.G.R., Khokhar N., Chen R., Basilico S. (2023). Dual targeting of CD19 and CD22 with bicistronic CAR-T cells in patients with relapsed/refractory large B-cell lymphoma. Blood.

[86] Wang T., Tang Y., Cai J., Wan X., Hu S., Lu X., Xie Z., Qiao X., Jiang H., Shao J. (2023). Coadministration of CD19- and CD22-Directed Chimeric Antigen Receptor T-Cell Therapy in Childhood B-Cell Acute Lymphoblastic Leukemia: A Single-Arm, Multicenter, Phase II Trial. J. Clin. Oncol..

[87] Kantarjian H.M., DeAngelo D.J., Stelljes M., Martinelli G., Liedtke M., Stock W., Gökbuget N., O’Brien S., Wang K., Wang T. (2016). Inotuzumab Ozogamicin versus Standard Therapy for Acute Lymphoblastic Leukemia. N. Engl. J. Med..

[88] Jabbour E., Short N.J., Senapati J., Jain N., Huang X., Daver N., DiNardo C.D., Pemmaraju N., Wierda W., Garcia-Manero G. (2023). Mini-hyper-CVD plus inotuzumab ozogamicin, with or without blinatumomab, in the subgroup of older patients with newly diagnosed Philadelphia chromosome-negative B-cell acute lymphocytic leukaemia: Long-term results of an open-label phase 2 trial. Lancet Haematol..

[89] He R.R., Nayer Z., Hogan M., Cuevo R.S., Woodward K., Heyer D., Curtis C.A., Peterson J.F. (2019). Immunotherapy- (Blinatumomab-) Related Lineage Switch of KMT2A/AFF1 Rearranged B-Lymphoblastic Leukemia into Acute Myeloid Leukemia/Myeloid Sarcoma and Subsequently into B/Myeloid Mixed Phenotype Acute Leukemia. Case Rep. Hematol..

[90] Wölfl M., Rasche M., Eyrich M., Schmid R., Reinhardt D., Schlegel P.G. (2018). Spontaneous reversion of a lineage switch following an initial blinatumomab-induced ALL-to-AML switch in MLL-rearranged infant ALL. Blood Adv..

[91] Haddox C.L., Mangaonkar A.A., Chen D., Shi M., He R., Oliveira J.L., Litzow M.R., Al-Kali A., Hogan W.J., Elliott M.A. (2017). Blinatumomab-induced lineage switch of B-ALL with t(4:11)(q21;q23) KMT2A/AFF1 into an aggressive AML: Pre- and post-switch phenotypic, cytogenetic and molecular analysis. Blood Cancer J..

[92] Fournier E., Inchiappa L., Delattre C., Pignon J.M., Danicourt F., Bemba M., Roche-Lestienne C., Daudignon A., Decool G., Roumier C. (2019). Increased risk of adverse acute myeloid leukemia after anti-CD19-targeted immunotherapies in KMT2A-rearranged acute lymphoblastic leukemia: A case report and review of the literature. Leuk. Lymphoma.

[93] Hoseini S.S., Espinosa-Cotton M., Guo H.F., Cheung N.V. (2020). Overcoming leukemia heterogeneity by combining T cell engaging bispecific antibodies. J. Immunother. Cancer.

[94] Jiménez-Morales S., Aranda-Uribe I.S., Pérez-Amado C.J., Ramírez-Bello J., Hidalgo-Miranda A. (2021). Mechanisms of Immunosuppressive Tumor Evasion: Focus on Acute Lymphoblastic Leukemia. Front. Immunol..

[95] Feucht J., Kayser S., Gorodezki D., Hamieh M., Döring M., Blaeschke F., Schlegel P., Bösmüller H., Quintanilla-Fend L., Ebinger M. (2016). T-cell responses against CD19+ pediatric acute lymphoblastic leukemia mediated by bispecific T-cell engager (BiTE) are regulated contrarily by PD-L1 and CD80/CD86 on leukemic blasts. Oncotarget.

[96] Kobayashi T., Ubukawa K., Fujishima M., Takahashi N. (2021). Correlation between increased immune checkpoint molecule expression and refractoriness to blinatumomab evaluated by longitudinal T cell analysis. Int. J. Hematol..

[97] Thompson P.A., Jiang X., Banerjee P., Basar R., Garg N., Chen K., Kaplan M., Nandivada V., Cortes A.K.N., Ferrajoli A. (2022). A phase two study of high dose blinatumomab in Richter’s syndrome. Leukemia.

[98] Sandhu K.S., Macias A., Del Real M., Beltran A.L., Kim Y.S., Zhang J., Palmer J., Robbins M., Loomis R., Akhtari M. (2022). Interim Results of a Phase 1/2 Study of Pembrolizumab Combined with Blinatumomab in Patients with Relapsed/Refractory (r/r) ALL. Blood.

[99] Webster J., Luskin M.R., Prince G.T., DeZern A.E., DeAngelo D.J., Levis M.J., Blackford A., Sharon E., Streicher H., Luznik L. (2018). Blinatumomab in Combination with Immune Checkpoint Inhibitors of PD-1 and CTLA-4 in Adult Patients with Relapsed/Refractory (R/R) CD19 Positive B-Cell Acute Lymphoblastic Leukemia (ALL): Preliminary Results of a Phase I Study. Blood.

[100] Armitage J.O., Gascoyne R.D., Lunning M.A., Cavalli F. (2017). Non-Hodgkin lymphoma. Lancet.

[101] Dotan E., Aggarwal C., Smith M.R. (2010). Impact of Rituximab (Rituxan) on the Treatment of B-Cell Non-Hodgkin’s Lymphoma. Pharm. Ther..

[102] Marofi F., Rahman H.S., Achmad M.H., Sergeevna K.N., Suksatan W., Abdelbasset W.K., Mikhailova M.V., Shomali N., Yazdanifar M., Hassanzadeh A. (2021). A Deep Insight into CAR-T Cell Therapy in Non-Hodgkin Lymphoma: Application, Opportunities, and Future Directions. Front. Immunol..

[103] Blanco B., Domínguez-Alonso C., Alvarez-Vallina L. (2021). Bispecific Immunomodulatory Antibodies for Cancer Immunotherapy. Clin. Cancer Res..

[104] Horna P., Nowakowski G., Endell J., Boxhammer R. (2019). Comparative Assessment of Surface CD19 and CD20 Expression on B-Cell Lymphomas from Clinical Biopsies: Implications for Targeted Therapies. Blood.

[105] Pavlasova G., Mraz M. (2020). The regulation and function of CD20: An “enigma” of B-cell biology and targeted therapy. Haematologica.

[106] Chung C. (2019). Current targeted therapies in lymphomas. Am. J. Health Syst. Pharm..

[107] Bartlett N.L., Sehn L.H., Matasar M.J., Schuster S.J., Assouline S., Giri P., Kuruvilla J., Canales M., Dietrich S., Fay K. (2022). Mosunetuzumab Monotherapy Demonstrates Durable Efficacy with a Manageable Safety Profile in Patients with Relapsed/Refractory Follicular Lymphoma Who Received ≥2 Prior Therapies: Updated Results from a Pivotal Phase II Study. Blood.

[108] Kim T.M., Taszner M., Cho S.-G., Novelli S., Le Gouill S., Poon M.L., Villasboas J.C., Champion R., Bachy E., Guidez S. (2022). Odronextamab in Patients with Relapsed/Refractory (R/R) Follicular Lymphoma (FL) Grade 1-3a: Results from a Prespecified Analysis of the Pivotal Phase II Study ELM-2. Blood.

[109] Falchi L., Carlo-Stella C., Morschhauser F., Hutchings M., Bachy E., Cartron G., Khan C., Tani M., Martinez-Lopez J., Bartlett N.L. (2023). Glofitamab monotherapy in pts with relapsed/refractory (R/R) large B-cell lymphoma (LBCL): Extended follow-up and landmark analyses from a pivotal phase II study. J. Clin. Oncol..

[110] Dickinson M.J., Carlo-Stella C., Morschhauser F., Bachy E., Corradini P., Iacoboni G., Khan C., Wróbel T., Offner F., Trněný M. (2022). Glofitamab for Relapsed or Refractory Diffuse Large B-Cell Lymphoma. N. Engl. J. Med..

[111] Thieblemont C., Phillips T., Ghesquieres H., Cheah C.Y., Clausen M.R., Cunningham D., Do Y.R., Feldman T., Gasiorowski R., Jurczak W. (2023). Epcoritamab, a Novel, Subcutaneous CD3xCD20 Bispecific T-Cell-Engaging Antibody, in Relapsed or Refractory Large B-Cell Lymphoma: Dose Expansion in a Phase I/II Trial. J. Clin. Oncol..

[112] Kim W.-S., Kim T.M., Cho S.-G., Jarque I., Iskierka-Jażdżewska E., Poon M.L., Prince H.M., Oh S.Y., Lim F., Carpio C. (2022). Odronextamab in Patients with Relapsed/Refractory (R/R) Diffuse Large B-Cell Lymphoma (DLBCL): Results from a Prespecified Analysis of the Pivotal Phase II Study ELM-2. Blood.

[113] Olszewski A.J., Avigdor A., Babu S., Levi I., Eradat H., Abadi U., Holmes H., McKinney M., Woszczyk D., Giannopoulos K. (2022). Mosunetuzumab Monotherapy Continues to Demonstrate Promising Efficacy and Durable Complete Responses in Elderly/Unfit Patients with Previously Untreated Diffuse Large B-Cell Lymphoma. Blood.

[114] Hutchings M., Mous R., Clausen M.R., Johnson P., Linton K.M., Chamuleau M.E.D., Lewis D.J., Sureda Balari A., Cunningham D., Oliveri R.S. (2021). Dose escalation of subcutaneous epcoritamab in patients with relapsed or refractory B-cell non-Hodgkin lymphoma: An open-label, phase 1/2 study. Lancet.

[115] Bachy E., Le Gouill S., Di Blasi R., Sesques P., Manson G., Cartron G., Beauvais D., Roulin L., Gros F.X., Rubio M.T. (2022). A real-world comparison of tisagenlecleucel and axicabtagene ciloleucel CAR T cells in relapsed or refractory diffuse large B cell lymphoma. Nat. Med..

[116] Budde E.L., Bartlett N.L., Giri P., Schuster S.J., Assouline S., Yoon S.-S., Fay K., Matasar M.J., Gutierrez N.C., Marlton P. (2022). Subcutaneous Mosunetuzumab Is Active with a Manageable Safety Profile in Patients (pts) with Relapsed/Refractory (R/R) B-Cell Non-Hodgkin Lymphomas (B-NHLs): Updated Results from a Phase I/II Study. Blood.

[117] Budde E., Gopal A.K., Kim W.S., Flinn I.W., Cheah C.Y.Y., Nastoupil L., Matasar M.J., Diefenbach C.S., Gregory G.P., Qazi I. (2021). A Phase 1 Dose Escalation Study of Igm-2323, a Novel Anti-CD20 x Anti-CD3 IgM T Cell Engager (TCE) in Patients with Advanced B-Cell Malignancies. Blood.

[118] Sermer D., Elavalakanar P., Abramson J.S., Palomba M.L., Salles G., Arnason J. (2023). Targeting CD19 for diffuse large B cell lymphoma in the era of CARs: Other modes of transportation. Blood Rev..

[119] Goebeler M.E., Knop S., Viardot A., Kufer P., Topp M.S., Einsele H., Noppeney R., Hess G., Kallert S., Mackensen A. (2016). Bispecific T-Cell Engager (BiTE) Antibody Construct Blinatumomab for the Treatment of Patients with Relapsed/Refractory Non-Hodgkin Lymphoma: Final Results from a Phase I Study. J. Clin. Oncol..

[120] Topp M., Dlugosz-Danecka M., Skotnicki A.B., Salogub G., Viardot A., Klein A.K., Hess G., Michel C.S., Grosicki S., Gural A. (2023). Safety of AFM11 in the treatment of patients with B-cell malignancies: Findings from two phase 1 studies. Trials.

[121] Nair R., Jacobs R., Cho S.-G., Devata S., Gaballa S., Yoon D.H., Stevens D.A., Kim J.S., Shah N.N., Brennan D.M. (2023). High complete response rate with TNB-486 in relapsed/refractory follicular lymphoma: Interim results from an ongoing phase 1 study. J. Clin. Oncol..

[122] Lesch S., Gill S. (2021). The promise and perils of immunotherapy. Blood Adv..

[123] Viardot A., Goebeler M.E., Hess G., Neumann S., Pfreundschuh M., Adrian N., Zettl F., Libicher M., Sayehli C., Stieglmaier J. (2016). Phase 2 study of the bispecific T-cell engager (BiTE) antibody blinatumomab in relapsed/refractory diffuse large B-cell lymphoma. Blood.

[124] Brody J., Wahlin B.E., Phillips T.J., Costello R., Lugtenburg P., Cordoba R., Wang L., Wu J., Elliott B., Abbas A. (2022). Epcoritamab (epco) with gemcitabine + oxaliplatin (GemOx) in patients (pts) with relapsed or refractory (R/R) diffuse large B-cell lymphoma (DLBCL) ineligible for autologous stem cell transplant (ASCT) induces high response rate even in pts failing CAR T therapy. J. Clin. Oncol..

[125] Parker K.R., Migliorini D., Perkey E., Yost K.E., Bhaduri A., Bagga P., Haris M., Wilson N.E., Liu F., Gabunia K. (2020). Single-Cell Analyses Identify Brain Mural Cells Expressing CD19 as Potential Off-Tumor Targets for CAR-T Immunotherapies. Cell.

[126] Rezvani A.R., Maloney D.G. (2011). Rituximab resistance. Best. Pract. Res. Clin. Haematol..

[127] Hayashi K., Nagasaki E., Kan S., Ito M., Kamata Y., Homma S., Aiba K. (2016). Gemcitabine enhances rituximab-mediated complement-dependent cytotoxicity to B cell lymphoma by CD20 upregulation. Cancer Sci..

[128] Hutchings M., Sureda A., Terol M.J., Bosch Albareda F., Corradini P., Larsen T.S., Rueda Dominguez A., Panchal A., Bottos A., Carlile D. (2021). Glofitamab (Glofit) in Combination with Polatuzumab Vedotin (Pola): Phase Ib/II Preliminary Data Support Manageable Safety and Encouraging Efficacy in Relapsed/Refractory (R/R) Diffuse Large B-Cell Lymphoma (DLBCL). Blood.

[129] Sehn L.H., Herrera A.F., Flowers C.R., Kamdar M.K., McMillan A., Hertzberg M., Assouline S., Kim T.M., Kim W.S., Ozcan M. (2020). Polatuzumab Vedotin in Relapsed or Refractory Diffuse Large B-Cell Lymphoma. J. Clin. Oncol..

[130] Bartlett N.L., Assouline S., Giri P., Schuster S.J., Cheah C.Y.Y., Matasar M.J., Gregory G.P., Yoon D.-H., Shadman M., Fay K. (2023). Mosunetuzumab monotherapy is active and tolerable in patients with relapsed/refractory diffuse large B-cell lymphoma. Blood Adv..

[131] Westin J., Olszewski A.J., Fogliatto L.M., Kim W.-S., Shin H.-J., Wu H., Yin S., Pham S., Penuel E., Jing J. (2022). SUNMO: A Phase III Trial Evaluating the Efficacy and Safety of Mosunetuzumab in Combination with Polatuzumab Vedotin Versus Rituximab in Combination with Gemcitabine Plus Oxaliplatin in Patients with Relapsed or Refractory Aggressive B-Cell Non-Hodgkin Lymphoma. Blood.

[132] Hertzberg M., Ku M., Catalani O., Althaus B., Simko S., Gregory G.P. (2021). A phase III trial evaluating glofitamab in combination with gemcitabine plus oxaliplatin versus rituximab in combination with gemcitabine and oxaliplatin in patients with relapsed/refractory (R/R) diffuse large B-cell lymphoma (DLBCL). J. Clin. Oncol..

[133] Engelberts P.J., Hiemstra I.H., de Jong B., Schuurhuis D.H., Meesters J., Beltran Hernandez I., Oostindie S.C., Neijssen J., van den Brink E.N., Horbach G.J. (2020). DuoBody-CD3xCD20 induces potent T-cell-mediated killing of malignant B cells in preclinical models and provides opportunities for subcutaneous dosing. EBioMedicine.

[134] Jiang Y., Li Y., Zhu B. (2015). T-cell exhaustion in the tumor microenvironment. Cell Death Dis..

[135] Piccione E.C., Belousov A., Hamidi H., Carlo-Stella C., Dickinson M., Morschhauser F., Lundberg L., Humphrey K., Tracy S., Hutchings M. (2022). P1210: Immune Correlates of Response to Glofitamab: Biomarker Findings from a Pivotal Phase Ii Expansion Study in Patients with Relapsed or Refractory (R/R) Diffuse Large B-Cell Lymphoma (Dlbcl). Hemasphere.

[136] Danhof S., Schreder M., Knop S., Rasche L., Strifler S., Löffler C., Gogishvili T., Einsele H., Hudecek M. (2018). Expression of programmed death-1 on lymphocytes in myeloma patients is lowered during lenalidomide maintenance. Haematologica.

[137] Merryman R., Belada D., Sureda A., Leppä S., Vermaat J.S.P., Holte H., Hutchings M., Lugtenburg P., de Vos S., Abrisqueta P. (2023). Epcoritamab + R2 regimen and responses in high-risk follicular lymphoma, regardless of POD24 status. J. Clin. Oncol..

[138] Morschhauser F., Bishton M., Eyre T.A., Bachy E., Cartron G., Ysebaert L., Bobillo S., Gutierrez N.C., Budde L.E., Fox C.P. (2021). Mosunetuzumab in Combination with Lenalidomide Has a Manageable Safety Profile and Encouraging Activity in Patients with Relapsed/Refractory Follicular Lymphoma: Initial Results from a Phase Ib Study. Blood.

[139] Falchi L., Morschhauser F., Gribben J.G., Huang H., Dinh M., Conlon R., Chen X., Elliot B., Seymour J.F. (2022). Phase 3 Trial of Subcutaneous Epcoritamab in Combination with Rituximab and Lenalidomide (R2) Vs R2 Among Patients with Relapsed or Refractory Follicular Lymphoma (EPCORE FL-1). Blood.

[140] Nastoupil L., Morschhauser F., Scholz C.W., Bishton M., Yoon S.S., Giri P., Wei M.C., Knapp A., Li C.C., Bottos A. (2022). P1125: Celestimo: A Phase Iii Trial Evaluating the Efficacy and Safety of Mosunetuzumab Plus Lenalidomide Versus Rituximab Plus Lenalidomide in Patients with Relapsed or Refractory Follicular Lymphoma. Hemasphere.

[141] Nastoupil L.J. (2022). CELESTIMO: A Randomized Phase III Trial Examining the Efficacy and Safety of Mosunetuzumab in Combination with Lenalidomide Versus Rituximab in Combination with Lenalidomide in Relapsed/Refractory Follicular Lymphoma. Hematologist.

[142] Smith-Garvin J.E., Koretzky G.A., Jordan M.S. (2009). T cell activation. Annu. Rev. Immunol..

[143] Chester C., Sanmamed M.F., Wang J., Melero I. (2018). Immunotherapy targeting 4-1BB: Mechanistic rationale, clinical results, and future strategies. Blood.

[144] Wei J., Montalvo-Ortiz W., Yu L., Krasco A., Olson K., Rizvi S., Fiaschi N., Coetzee S., Wang F., Ullman E. (2022). CD22-targeted CD28 bispecific antibody enhances antitumor efficacy of odronextamab in refractory diffuse large B cell lymphoma models. Sci. Transl. Med..

[145] Hutchings M., Carlo-Stella C., Gritti G., Bosch F., Morschhauser F., Townsend W., Offner F., Walter H.S., Ghesquieres H., Houot R. (2022). CD19 4-1BBL (RO7227166) a Novel Costimulatory Bispecific Antibody Can be Safely Combined with the T-Cell-Engaging Bispecific Antibody Glofitamab in Relapsed or Refractory B-Cell Non-Hodgkin Lymphoma. Blood.

[146] Döhner H., Weisdorf D.J., Bloomfield C.D. (2015). Acute Myeloid Leukemia. N. Engl. J. Med..

[147] Mohamed Jiffry M.Z., Kloss R., Ahmed-Khan M., Carmona-Pires F., Okam N., Weeraddana P., Dharmaratna D., Dandwani M., Moin K. (2023). A review of treatment options employed in relapsed/refractory AML. Hematology.

[148] Guy D.G., Uy G.L. (2018). Bispecific Antibodies for the Treatment of Acute Myeloid Leukemia. Curr. Hematol. Malig. Rep..

[149] Liu J., Tong J., Yang H. (2022). Targeting CD33 for acute myeloid leukemia therapy. BMC Cancer.

[150] Krupka C., Kufer P., Kischel R., Zugmaier G., Bögeholz J., Köhnke T., Lichtenegger F.S., Schneider S., Metzeler K.H., Fiegl M. (2014). CD33 target validation and sustained depletion of AML blasts in long-term cultures by the bispecific T-cell-engaging antibody AMG 330. Blood.

[151] Pollard J.A., Alonzo T.A., Loken M., Gerbing R.B., Ho P.A., Bernstein I.D., Raimondi S.C., Hirsch B., Franklin J., Walter R.B. (2012). Correlation of CD33 expression level with disease characteristics and response to gemtuzumab ozogamicin containing chemotherapy in childhood AML. Blood.

[152] Taussig D.C., Pearce D.J., Simpson C., Rohatiner A.Z., Lister T.A., Kelly G., Luongo J.L., Danet-Desnoyers G.A., Bonnet D. (2005). Hematopoietic stem cells express multiple myeloid markers: Implications for the origin and targeted therapy of acute myeloid leukemia. Blood.

[153] Jen E.Y., Ko C.W., Lee J.E., Del Valle P.L., Aydanian A., Jewell C., Norsworthy K.J., Przepiorka D., Nie L., Liu J. (2018). FDA Approval: Gemtuzumab Ozogamicin for the Treatment of Adults with Newly Diagnosed CD33-Positive Acute Myeloid Leukemia. Clin. Cancer Res..

[154] Yu B., Liu D. (2019). Gemtuzumab ozogamicin and novel antibody-drug conjugates in clinical trials for acute myeloid leukemia. Biomark. Res..

[155] Espinoza-Gutarra M.R., Green S.D., Zeidner J.F., Konig H. (2021). CD123-targeted therapy in acute myeloid leukemia. Expert. Rev. Hematol..

[156] Muñoz L., Nomdedéu J.F., López O., Carnicer M.J., Bellido M., Aventín A., Brunet S., Sierra J. (2001). Interleukin-3 receptor alpha chain (CD123) is widely expressed in hematologic malignancies. Haematologica.

[157] Testa U., Riccioni R., Militi S., Coccia E., Stellacci E., Samoggia P., Latagliata R., Mariani G., Rossini A., Battistini A. (2002). Elevated expression of IL-3Ralpha in acute myelogenous leukemia is associated with enhanced blast proliferation, increased cellularity, and poor prognosis. Blood.

[158] Ravandi F., Bashey A., Stock W., Foran J.M., Mawad R., Egan D., Blum W., Yang A., Pastore A., Johnson C. (2020). Complete Responses in Relapsed/Refractory Acute Myeloid Leukemia (AML) Patients on a Weekly Dosing Schedule of Vibecotamab (XmAb14045), a CD123 x CD3 T Cell-Engaging Bispecific Antibody; Initial Results of a Phase 1 Study. Blood.

[159] Watts J., Maris M., Lin T.L., Patel P., Madanat Y.F., Cogle C.R., Borthakur G., Huebner D., Khaskhely N., Bonham L. (2022). Updated Results from a Phase 1 Study of APVO436, a Novel Bispecific Anti-CD123 x Anti-CD3 Adaptir™ Molecule, in Relapsed/Refractory Acute Myeloid Leukemia and Myelodysplastic Syndrome. Blood.

[160] Winer E.S., Maris M., Sharma M.R., Kaminker P., Zhao E., Ward A., Sochacki A.L. (2022). A Phase 1, First-in-Human, Dose-Escalation Study of MGD024, a CD123 x CD3 Bispecific Dart® Molecule, in Patients with Relapsed or Refractory CD123-Positive (+) Hematologic Malignancies. Blood.

[161] Ma H., Padmanabhan I.S., Parmar S., Gong Y. (2019). Targeting CLL-1 for acute myeloid leukemia therapy. J. Hematol. Oncol..

[162] Bakker A.B., van den Oudenrijn S., Bakker A.Q., Feller N., van Meijer M., Bia J.A., Jongeneelen M.A., Visser T.J., Bijl N., Geuijen C.A. (2004). C-type lectin-like molecule-1: A novel myeloid cell surface marker associated with acute myeloid leukemia. Cancer Res..

[163] van Rhenen A., van Dongen G.A., Kelder A., Rombouts E.J., Feller N., Moshaver B., Stigter-van Walsum M., Zweegman S., Ossenkoppele G.J., Jan Schuurhuis G. (2007). The novel AML stem cell associated antigen CLL-1 aids in discrimination between normal and leukemic stem cells. Blood.

[164] Van Loo P.F., Doornbos R., Dolstra H., Shamsili S., Bakker L. (2015). Preclinical Evaluation of MCLA117, a CLEC12AxCD3 Bispecific Antibody Efficiently Targeting a Novel Leukemic Stem Cell Associated Antigen in AML. Blood.

[165] van Loo P.F., Hangalapura B.N., Thordardottir S., Gibbins J.D., Veninga H., Hendriks L.J.A., Kramer A., Roovers R.C., Leenders M., de Kruif J. (2019). MCLA-117, a CLEC12AxCD3 bispecific antibody targeting a leukaemic stem cell antigen, induces T cell-mediated AML blast lysis. Expert. Opin. Biol. Ther..

[166] Levis M., Small D. (2003). FLT3: ITDoes matter in leukemia. Leukemia.

[167] Kuchenbauer F., Kern W., Schoch C., Kohlmann A., Hiddemann W., Haferlach T., Schnittger S. (2005). Detailed analysis of FLT3 expression levels in acute myeloid leukemia. Haematologica.

[168] Durben M., Schmiedel D., Hofmann M., Vogt F., Nübling T., Pyz E., Bühring H.J., Rammensee H.G., Salih H.R., Große-Hovest L. (2015). Characterization of a bispecific FLT3 X CD3 antibody in an improved, recombinant format for the treatment of leukemia. Mol. Ther..

[169] Zhao J.C., Agarwal S., Ahmad H., Amin K., Bewersdorf J.P., Zeidan A.M. (2022). A review of FLT3 inhibitors in acute myeloid leukemia. Blood Rev..

[170] Antar A.I., Otrock Z.K., Jabbour E., Mohty M., Bazarbachi A. (2020). FLT3 inhibitors in acute myeloid leukemia: Ten frequently asked questions. Leukemia.

[171] Hofmann M., Große-Hovest L., Nübling T., Pyż E., Bamberg M.L., Aulwurm S., Bühring H.J., Schwartz K., Haen S.P., Schilbach K. (2012). Generation, selection and preclinical characterization of an Fc-optimized FLT3 antibody for the treatment of myeloid leukemia. Leukemia.

[172] Mehta N.K., Pfluegler M., Meetze K., Li B., Sindel I., Vogt F., Marklin M., Heitmann J.S., Kauer J., Osburg L. (2022). A novel IgG-based FLT3xCD3 bispecific antibody for the treatment of AML and B-ALL. J. Immunother. Cancer.

[173] Leclercq G., Steinhoff N., Haegel H., De Marco D., Bacac M., Klein C. (2022). Novel strategies for the mitigation of cytokine release syndrome induced by T cell engaging therapies with a focus on the use of kinase inhibitors. Oncoimmunology.

[174] Lamba J.K., Chauhan L., Shin M., Loken M.R., Pollard J.A., Wang Y.C., Ries R.E., Aplenc R., Hirsch B.A., Raimondi S.C. (2017). CD33 Splicing Polymorphism Determines Gemtuzumab Ozogamicin Response in De Novo Acute Myeloid Leukemia: Report from Randomized Phase III Children’s Oncology Group Trial AAML0531. J. Clin. Oncol..

[175] Isidori A., Salvestrini V., Ciciarello M., Loscocco F., Visani G., Parisi S., Lecciso M., Ocadlikova D., Rossi L., Gabucci E. (2014). The role of the immunosuppressive microenvironment in acute myeloid leukemia development and treatment. Expert. Rev. Hematol..

[176] Lamble A.J., Lind E.F. (2018). Targeting the Immune Microenvironment in Acute Myeloid Leukemia: A Focus on T Cell Immunity. Front. Oncol..

[177] Tettamanti S., Pievani A., Biondi A., Dotti G., Serafini M. (2022). Catch me if you can: How AML and its niche escape immunotherapy. Leukemia.

[178] Wang H., Tao Q., Wang Z., Zhang Q., Xiao H., Zhou M., Dong Y., Zhai Z. (2020). Circulating Monocytic Myeloid-Derived Suppressor Cells Are Elevated and Associated with Poor Prognosis in Acute Myeloid Leukemia. J. Immunol. Res..

[179] Lv M., Wang K., Huang X.J. (2019). Myeloid-derived suppressor cells in hematological malignancies: Friends or foes. J. Hematol. Oncol..

[180] Dong Y., Han Y., Huang Y., Jiang S., Huang Z., Chen R., Yu Z., Yu K., Zhang S. (2020). PD-L1 Is Expressed and Promotes the Expansion of Regulatory T Cells in Acute Myeloid Leukemia. Front. Immunol..

[181] Zhou Q., Munger M.E., Highfill S.L., Tolar J., Weigel B.J., Riddle M., Sharpe A.H., Vallera D.A., Azuma M., Levine B.L. (2010). Program death-1 signaling and regulatory T cells collaborate to resist the function of adoptively transferred cytotoxic T lymphocytes in advanced acute myeloid leukemia. Blood.

[182] Yang X., Ma L., Zhang X., Huang L., Wei J. (2022). Targeting PD-1/PD-L1 pathway in myelodysplastic syndromes and acute myeloid leukemia. Exp. Hematol. Oncol..

[183] Cheng P., Chen X., Dalton R., Calescibetta A., So T., Gilvary D., Ward G., Smith V., Eckard S., Fox J.A. (2022). Immunodepletion of MDSC by AMV564, a novel bivalent, bispecific CD33/CD3 T cell engager, ex vivo in MDS and melanoma. Mol. Ther..

[184] Cheng P., Eksioglu E., Chen X., Wei M., Guenot J., Fox J., List A.F., Wei S. (2017). Immunodepletion of MDSC By AMV564, a Novel Tetravalent Bispecific CD33/CD3 T Cell Engager Restores Immune Homeostasis in MDS in Vitro. Blood.

[185] Jitschin R., Saul D., Braun M., Tohumeken S., Völkl S., Kischel R., Lutteropp M., Dos Santos C., Mackensen A., Mougiakakos D. (2018). CD33/CD3-bispecific T-cell engaging (BiTE^®^) antibody construct targets monocytic AML myeloid-derived suppressor cells. J. Immunother. Cancer.

[186] Cosenza M., Sacchi S., Pozzi S. (2021). Cytokine Release Syndrome Associated with T-Cell-Based Therapies for Hematological Malignancies: Pathophysiology, Clinical Presentation, and Treatment. Int. J. Mol. Sci..

[187] Leclercq G., Servera L.A., Danilin S., Challier J., Steinhoff N., Bossen C., Odermatt A., Nicolini V., Umaña P., Klein C. (2022). Dissecting the mechanism of cytokine release induced by T-cell engagers highlights the contribution of neutrophils. Oncoimmunology.

[188] Shimabukuro-Vornhagen A., Gödel P., Subklewe M., Stemmler H.J., Schlößer H.A., Schlaak M., Kochanek M., Böll B., von Bergwelt-Baildon M.S. (2018). Cytokine release syndrome. J. Immunother. Cancer.

[189] Lee D.W., Santomasso B.D., Locke F.L., Ghobadi A., Turtle C.J., Brudno J.N., Maus M.V., Park J.H., Mead E., Pavletic S. (2019). ASTCT Consensus Grading for Cytokine Release Syndrome and Neurologic Toxicity Associated with Immune Effector Cells. Biol. Blood Marrow Transplant..

[190] Sharma S., Wang D., Lon H.-K., Soltantabar P., Viqueira A., Czibere A., Hickman A., White J.L., Elmeliegy M. (2022). A Systematic Meta-Analysis of Cytokine Release Syndrome Incidence in B-Cell Maturation Antigen-Targeting Chimeric Antigen Receptor T-Cell Therapy and Bispecific Antibodies for Patients with Relapsed and/or Refractory Multiple Myeloma. Blood.

[191] Li J., Piskol R., Ybarra R., Chen Y.J., Li J., Slaga D., Hristopoulos M., Clark R., Modrusan Z., Totpal K. (2019). CD3 bispecific antibody-induced cytokine release is dispensable for cytotoxic T cell activity. Sci. Transl. Med..

[192] Ball K., Dovedi S.J., Vajjah P., Phipps A. (2023). Strategies for clinical dose optimization of T cell-engaging therapies in oncology. MAbs.

[193] Trudel S., Bahlis N.J., Spencer A., Kaedbey R., Rodriguez Otero P., Harrison S.J., Wong C., Goodman G.R., Nakamura R., Choeurng V. (2022). Pretreatment with Tocilizumab Prior to the CD3 Bispecific Cevostamab in Patients with Relapsed/Refractory Multiple Myeloma (RRMM) Showed a Marked Reduction in Cytokine Release Syndrome Incidence and Severity. Blood.

[194] Mohan M., Nagavally S., Dhakal B., Radhakrishnan S.V., Chhabra S., D’Souza A., Hari P. (2022). Risk of infections with B-cell maturation antigen-directed immunotherapy in multiple myeloma. Blood Adv..

[195] Mazahreh F., Mazahreh L., Schinke C., Thanendrarajan S., Zangari M., Shaughnessy J.D., Zhan F., van Rhee F., Al Hadidi S. (2023). Risk of infections associated with the use of bispecific antibodies in multiple myeloma: A pooled analysis. Blood Adv..

[196] Lancman G., Parsa K., Rodriguez C., Richter J., Cho H.J., Parekh S., Richard S., Rossi A., Sanchez L., Thibaud S. (2022). Infections and Severe Hypogammaglobulinemia in Multiple Myeloma Patients Treated with Anti-BCMA Bispecific Antibodies. Blood.

[197] Hammons L.R., Szabo A., Janardan A., Dhakal B., Chhabra S., D’Souza A., Mohan M. (2022). Kinetics of Humoral Immunodeficiency with Bispecific Antibody Therapy in Relapsed Refractory Multiple Myeloma. JAMA Netw. Open.

[198] Moreau P., Touzeau C. (2022). T-cell-redirecting bispecific antibodies in multiple myeloma: A revolution?. Blood.

[199] Juluri K.R., Wu Q.V., Voutsinas J., Hou J., Hirayama A.V., Mullane E., Miles N., Maloney D.G., Turtle C.J., Bar M. (2022). Severe cytokine release syndrome is associated with hematologic toxicity following CD19 CAR T-cell therapy. Blood Adv..

[200] Morris E.C., Neelapu S.S., Giavridis T., Sadelain M. (2022). Cytokine release syndrome and associated neurotoxicity in cancer immunotherapy. Nat. Rev. Immunol..

